# Supplementary material for: Cell-inspired design of cascade catalysis system by 3D spatially separated active sites
Source: Nat Commun. 2023 Sep 2;14:5338. doi: 10.1038/s41467-023-41002-5 (PMC10475024; doi:10.1038/s41467-023-41002-5)
Supplement: Supplementary file 1 — Supplementary Information [file 41467_2023_41002_MOESM1_ESM.pdf]

Supplementary Information

Cell-inspired design of cascade catalysis system by 3D spatially separated active sites

Wang *et al.*

Supplementary Figures and Tables (2-36)

Supplementary Methods (37)

Supplementary References (38-39)

Supplementary Figures and Tables

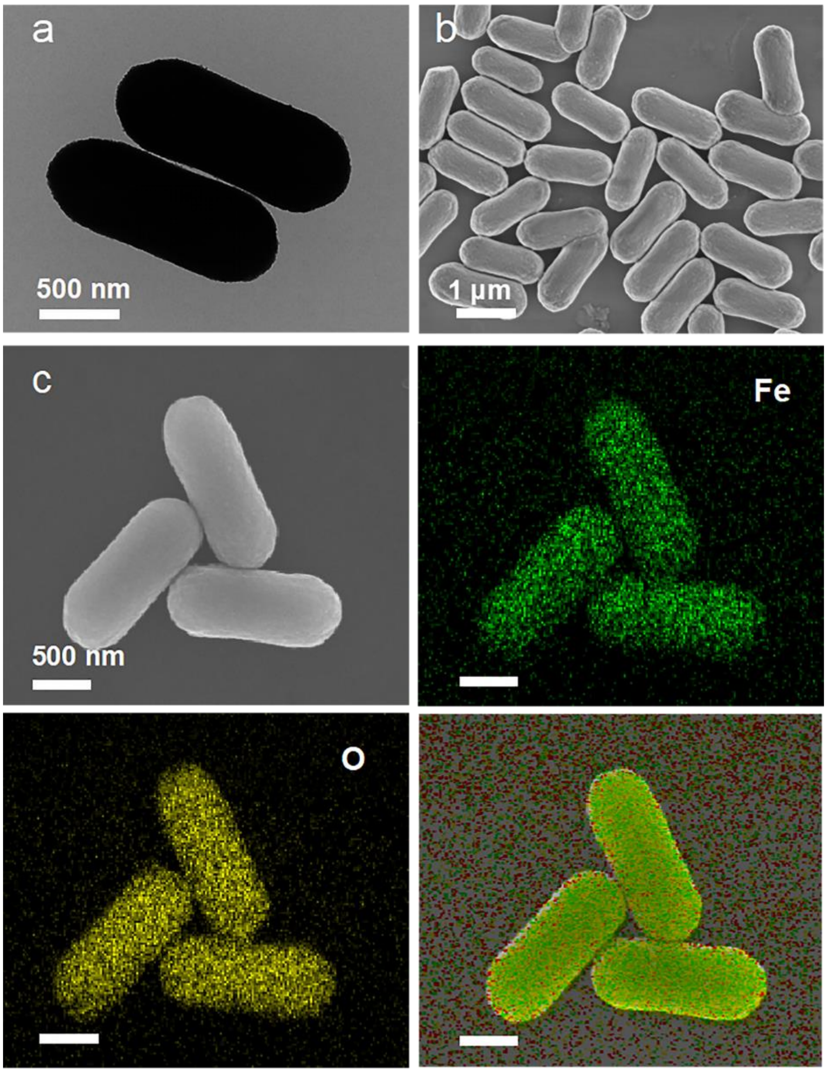

Supplementary Figure 1. Morphology characterization of Fe_2O_3. (a) TEM image, (b) SEM image, and (c) EDS mappings of Fe_2O_3. Note: TEM and SEM images show the Fe_2O_3 with peanut-shaped morphology. EDS mappings show the Fe and O were homogeneously dispersed over the whole architecture.

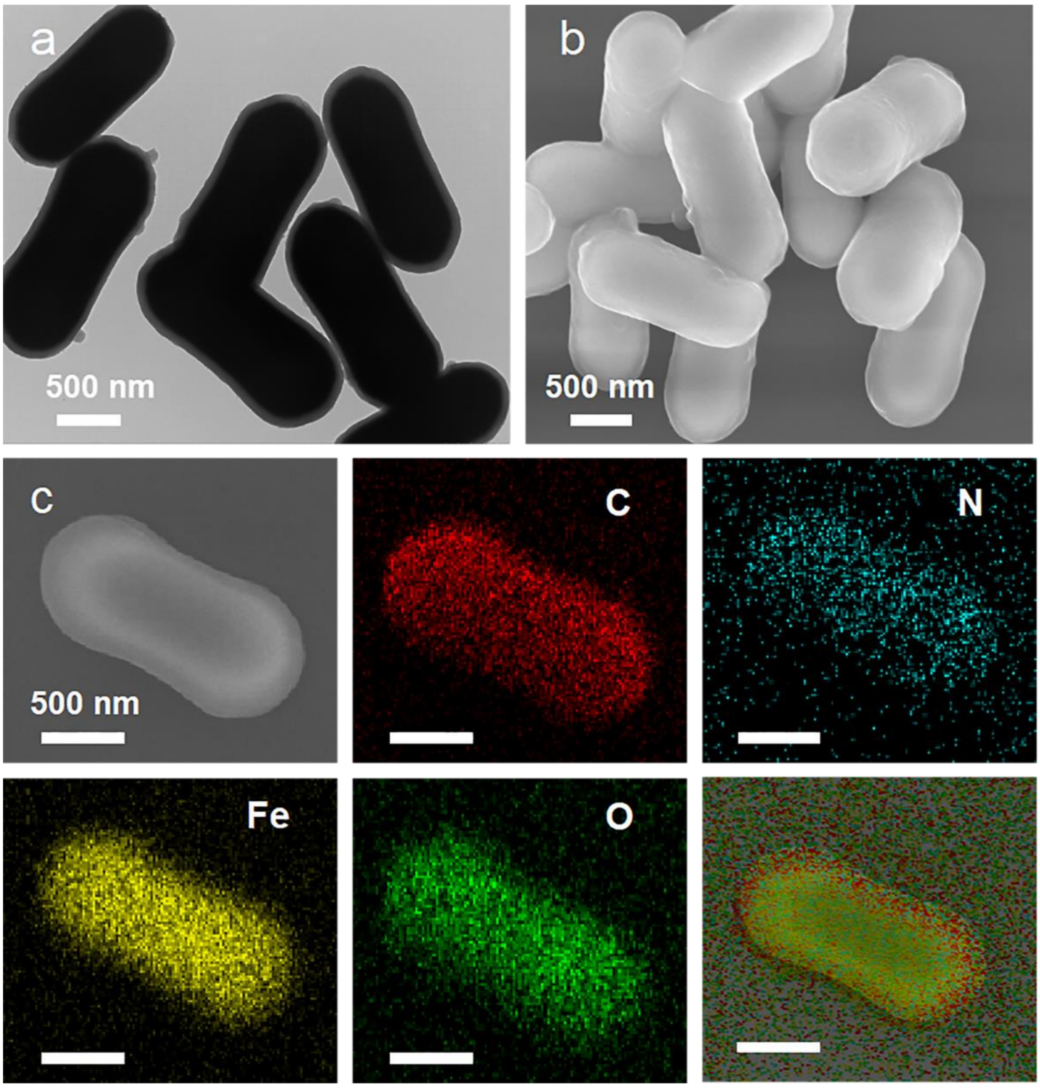

Supplementary Figure 2. Morphology characterization of Fe_2O_3@PDA. (a) TEM image, (b) SEM image, and (c) EDS mappings of Fe_2O_3@PDA. Note: TEM and SEM images show the core–shell architecture Fe_2O_3@PDA with retained peanut-shaped morphology. EDS mappings show the C, N and O were homogeneously dispersed over the whole architecture, whereas the Fe is mainly concentrated in the core of Fe_2O_3@PDA.

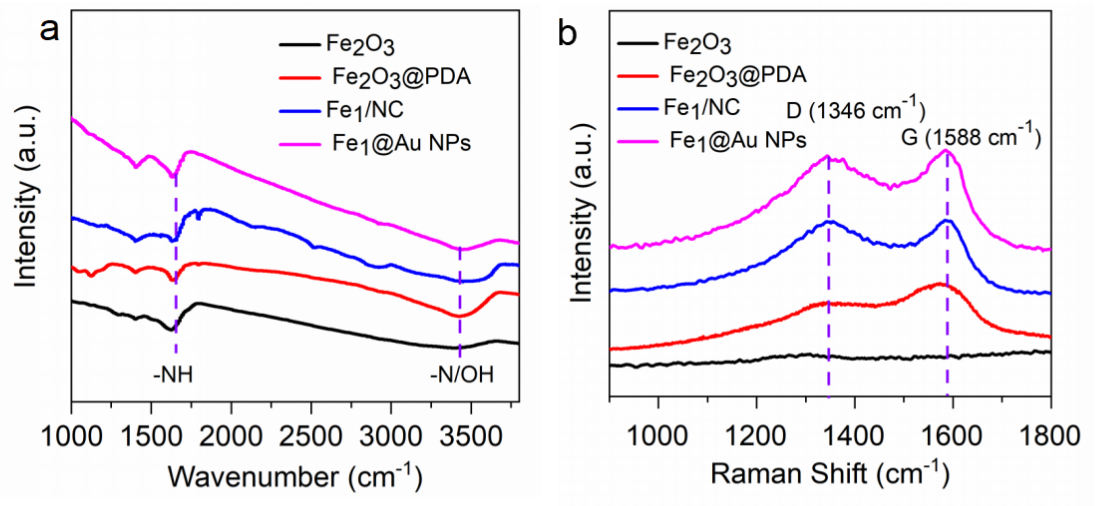

Supplementary Figure 3. Structure characterization of Fe_2O_3, Fe_2O_3@PDA, Fe_1/NC and Fe_1@Au NPs. (a) FT-IR spectra and (b) Raman spectra. Note: FT-IR spectroscopy presented in Supplementary Fig. 3a shows a typical stretching vibration for N/O–H at 3450 cm^{-1} and a bending vibration of N–H at 1615 cm^{-1} after coating the polydopaminelayer, indicating the successful construction of Fe_2O_3@PDA core–shell architecture. D (1346cm^{-1}) and G (1588 cm^{-1}) bands appeared after carbonization, indicating the successful transformation from PDA layers and other organic molecules to carbon species. In addition, the broad peak of D band with relatively high intensity and the high ID/IG value (≈ 0.98) indicates the generated disorder carbon.

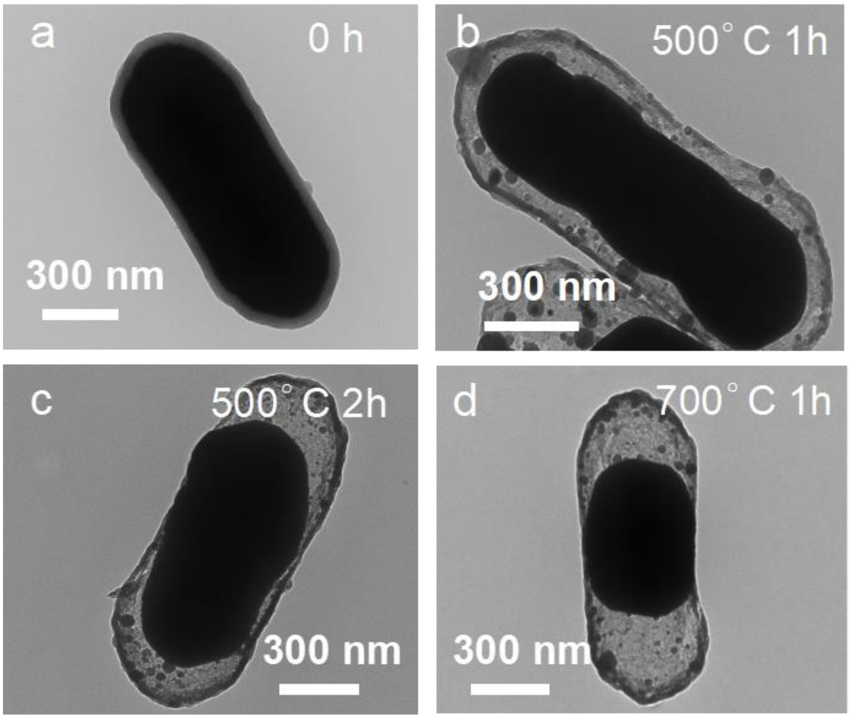

Supplementary Figure 4. TEM images of Fe_1/NC at different pyrolysis temperature and times. Note: To further elucidate the intrinsic mechanism of thermal atomization, TEM was performed to record the evolution process. As shown in Supplementary Fig. 4, the core–shell architecture was existed in Fe_2O_3@PDA initially. However, after carbonization at 500°C for 1 h, the PDA layer was transformed in situ to form N-doped carbon (NC) shells. The core with diameter (in length) of 600 nm can be observed and its diameter was decreased to 484 nm at 2 h, rapidly reduced to 276 nm in the next 1 h (700°C). Meanwhile, the core of Fe_2O_3 is gradually reduced to metal Fe by carbon, accompanied with the Fe atoms diffused locally in the heat drive and trapped by N defects on NC shells.

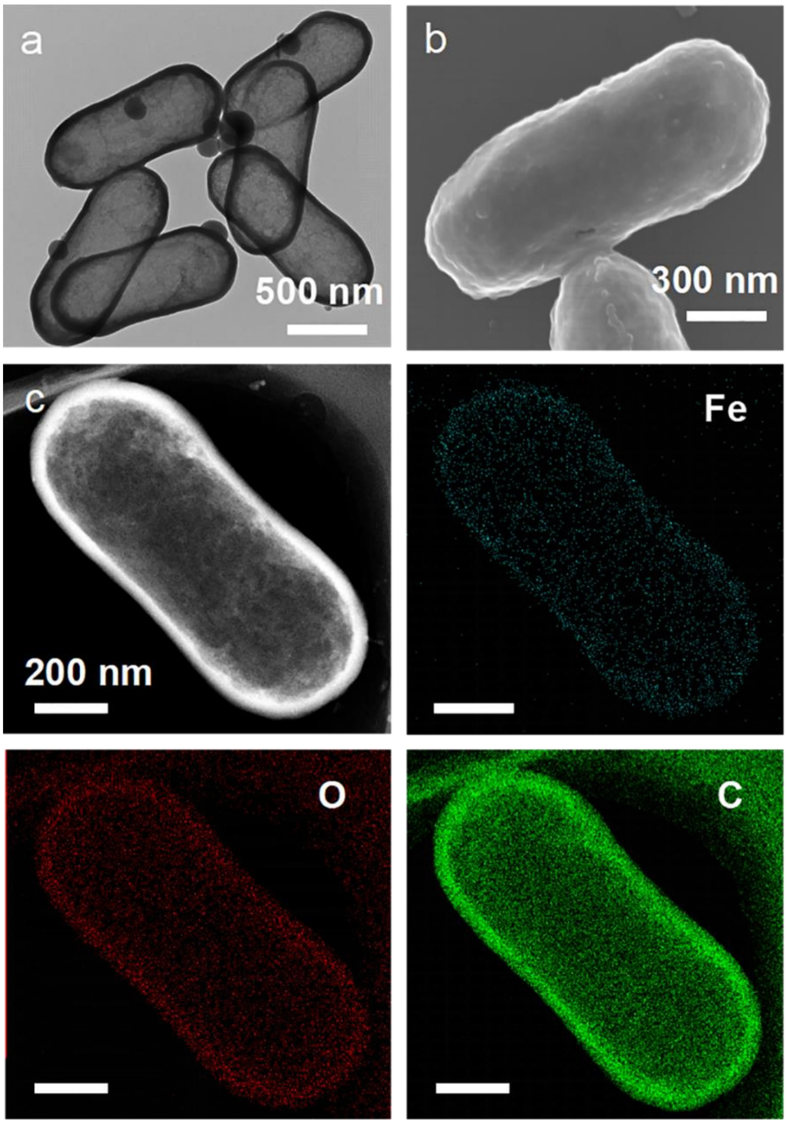

Supplementary Figure 5. Morphology characterization of Fe_1/NC. (a) TEM image, (b) SEM image, and (c) EDS mappings of Fe_1/NC. Note: TEM and SEM images show the obtained hollow Fe_1/NC with retained peanut-shaped morphology. EDS mappings show the C and O were homogeneously dispersed over the NC shells, whereas the Fe is mainly concentrated in the inner surface of NC shells.

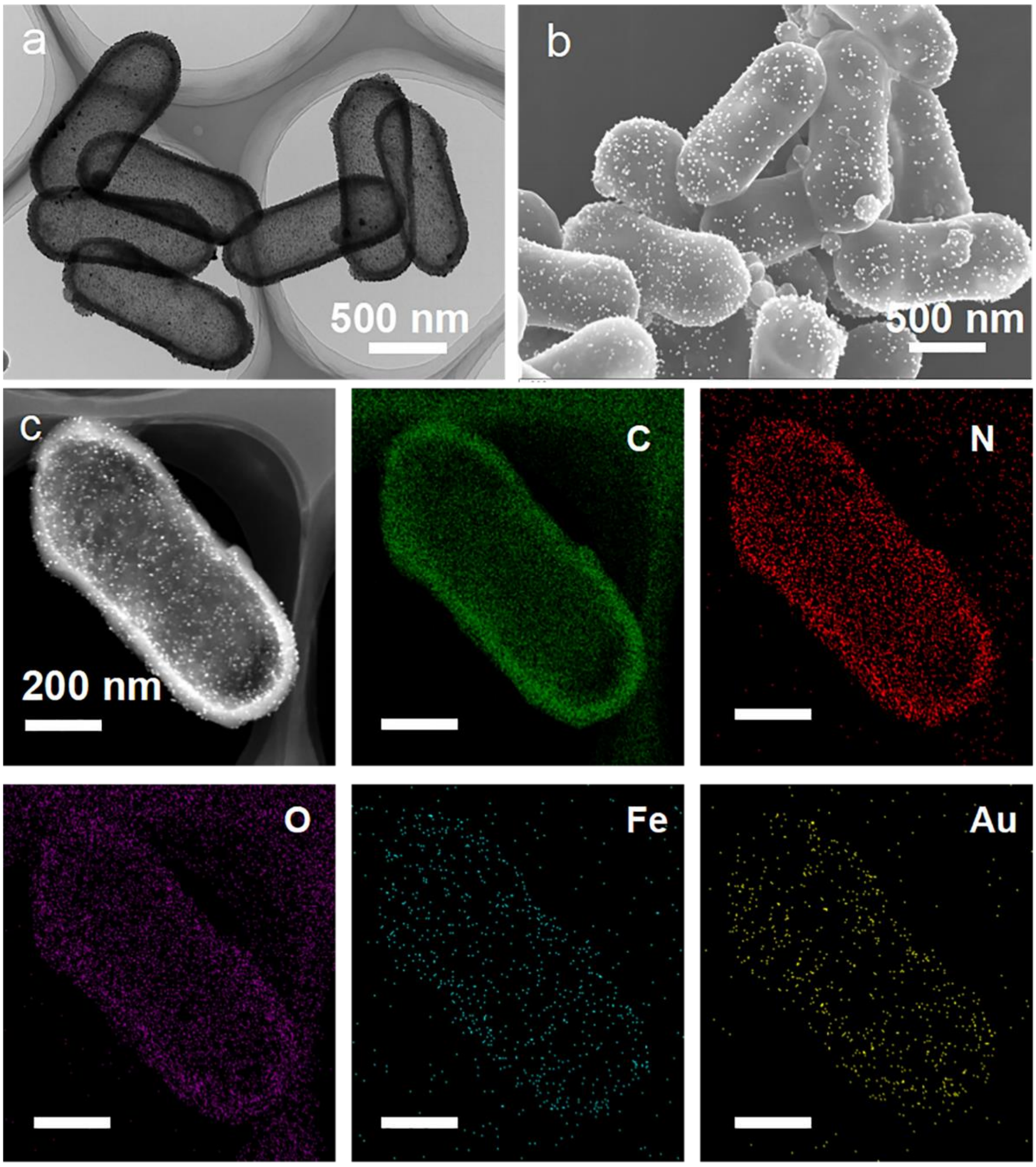

Supplementary Figure 6. Morphology characterization of Fe_1@Au NPs. (a) TEM image, (b) SEM image, and (c) EDS mappings of Fe_1@Au NPs. Note: TEM and SEM images show the obtained Fe_1/Au NPs with retained peanut-shaped morphology. As shown in Supplementary Fig. 6b, the Au NPs were homogeneously dispersed on the outer surface of NC shells. EDS mappings show the C, N, and O were homogeneously dispersed over the NC shells, whereas the Fe and Au are mainly concentrated on the inner and outer surfaces of NC shells, respectively.

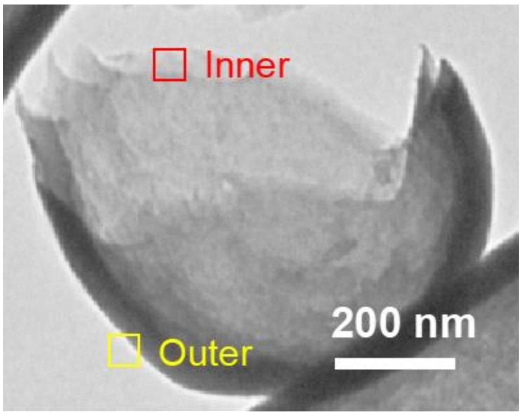

Supplementary Figure 7. TEM image of Fe_1/NC. Inset: The red and yellow boxes represent the inner and outer surface of NC shells, respectively. Note: Fig. 1f -g are the magnified HAADF-STEM images of the red and yellow boxes in Supplementary Fig. 7, respectively.

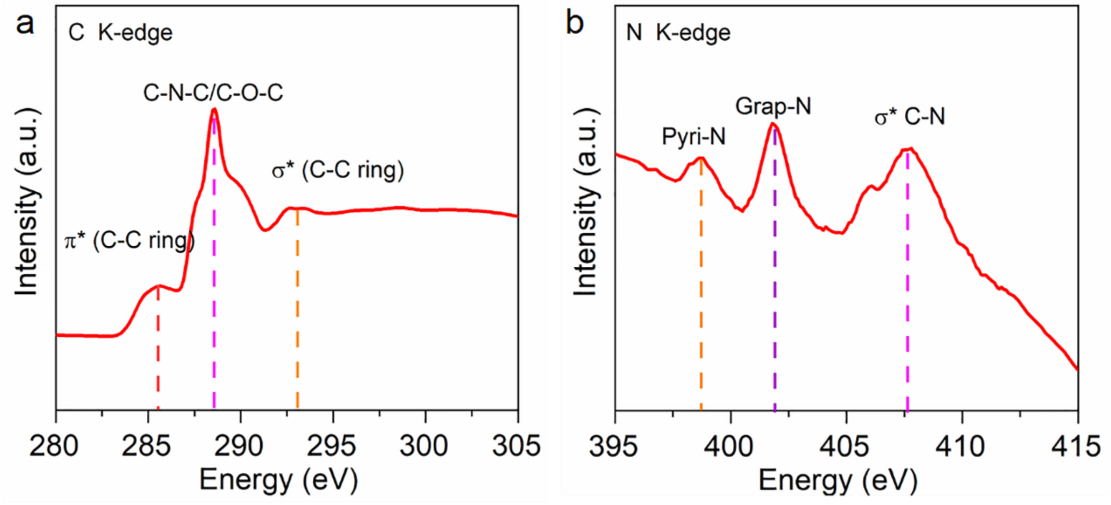

Supplementary Figure 8. Structure characterization of Fe_1@Au NPs. The NEXAFS spectra at the (a) C K-edge and (b) N K-edge. Note: The near edge X-ray absorption fine structure (NEXAFS) of the C k-edge shows peaks at 285.6 and 292.8 eV are due to C-C π* (ring) excitation and C-C σ* (ring) transitions, revealing that the graphited structure of Fe_1@Au NPs. The peak at 288.5 eV corresponding to defects in the carbon lattice including vacancies, bending and distortion for Fe_1@Au NPs resulted from pyrolysis effect.[1] In addition, the N k-edge shows π* resonance peaks is interpreted as pyridinic N at 398.6 eV, and graphitic N at 401.6 eV, respectively, which is consistent with the N 1*s* XPS spectra.

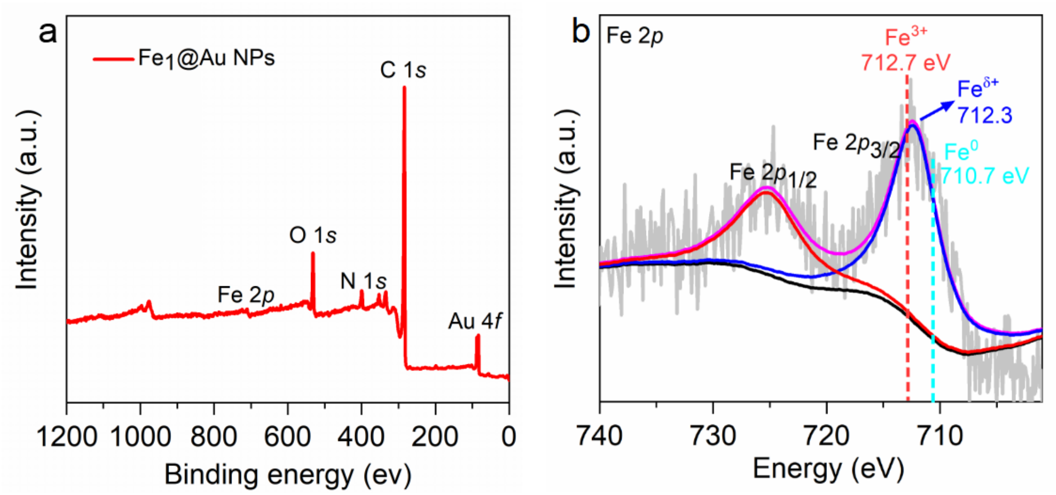

Supplementary Figure 9. XPS spectra of Fe_1@Au NPs. (a) The survey spectrum and (b) XPS Fe 2*p* spectrum. Note: The high-resolution XPS spectra show that the binding energy of Fe $2p_{3/2}$ peak centers at 712.3 eV (close to Fe^{3+}), revealing the ionic Fe δ^+ ($\delta \approx 3$) nature of Fe atoms.

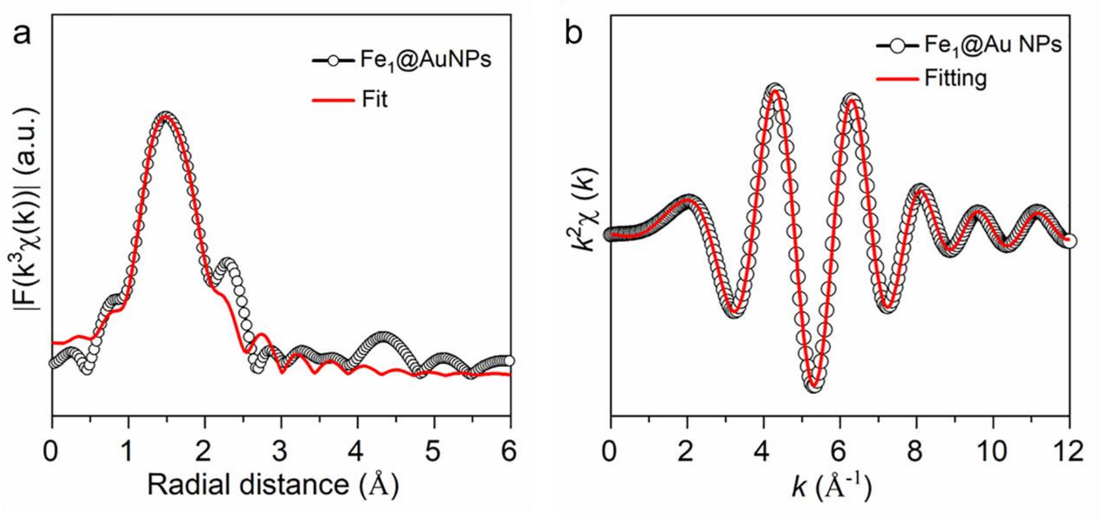

Supplementary Figure 10. EXAFS fitting curves of Fe_1@Au NPs. (a) R space and (b) k-space.

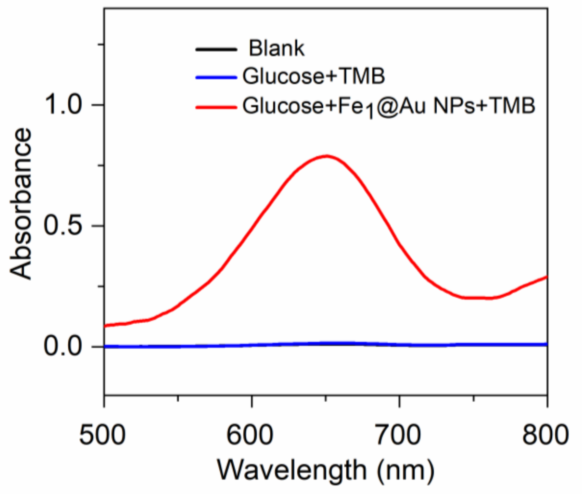

Supplementary Figure 11. The UV-Vis absorption spectra of solutions obtained by HRP-based colorimetric assay. Black line: blank solution containing HRP and TMB. Blue line: the solution obtained by reaction of HRP and TMB with glucose. Red line: the solution obtained by reaction of the mixture of HRP and TMB with the solution after the reaction of glucose and Fe_1@Au NPs and then centrifuge to remove the Fe_1@Au NPs. Note: The GOx-mimicking activity of biomimetic Fe_1@Au NPs system was evaluated. The obtained biomimetic Fe_1@Au NPs system can catalyze the oxidation of glucose to gluconic acid and H_2O_2 in the presence of O_2. After centrifuging the reaction solution to remove the Fe_1@Au NPs, the formed gluconic acid in the supernatant was verified by a specific colorimetric assay.[2-4] When hydroxylamine (NH_2OH) and $FeCl_3$ were successively introduced into the supernatant, leading to the formation of a red compound hydroxamate-Fe^{3+} with a distinct absorbance peak at 505 nm,[5] which confirms the production of gluconic acid in Fe_1@Au NPs-catalyzed cascade reaction (Fig. 3a). The other product of the glucose oxidation reaction, H_2O_2, was detected using the HRP-based colorimetric assay.[6] As shown in Supplementary Fig. 11, the reaction solution exhibits a characteristic absorbance of the oxidative product of 3,3',5,5'-tetramethylbenzidine (TMB) at 652 nm when HRP and TMB were added to the supernatant, suggesting the generation of H_2O_2 and the existence of GOx-mimicking activity in biomimetic Fe_1@Au NPs system.

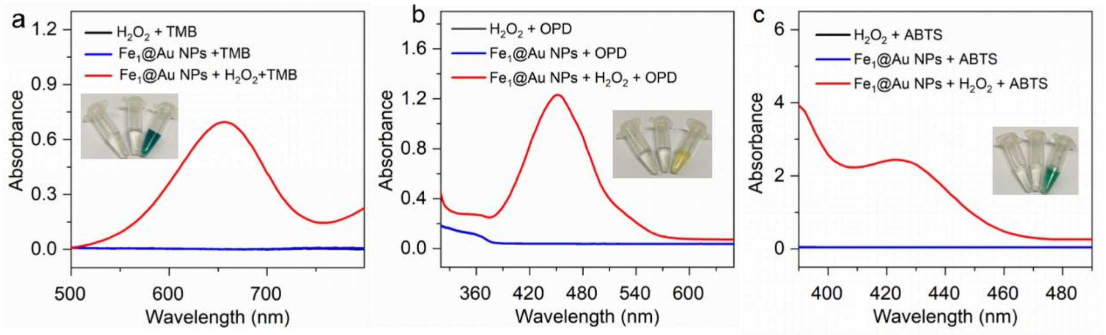

Supplementary Figure 12. The typical UV/Vis spectra of different reaction systems. (a) The reaction systems with TMB, (b) OPD and (c) ABTS as peroxidase substrates (inset: optical image showing the corresponding color changes). Note: To evaluate the POD-mimicking activity of biomimetic Fe_1@Au NPs system, we carried out a typical chromogenic reaction by catalytic oxidation of TMB in the presence of H_2O_2.[7] As shown in Supplementary Fig.12a, Fe_1@Au NPs can catalyze the oxidation of TMB to generate the blue oxTMB with the distinct absorption peak at 652 nm. Negligible absorbance in the UV-Vis absorption spectra was observed in Fe_1@Au NPs/TMB and H_2O_2/TMB, indicating the peroxidase activity of biomimetic Fe_1@Au NPs system. Besides, the *o*-phenylenediamine (OPD) and 2, 2′-azino-bis(3-ethylbenzothiazoline-6-sulfonic acid) (ABTS) were also selected as the chromogenic substrates. As shown in Supplementary Fig.12b-c, Fe_1@Au NPs can catalyze oxidation OPD to give an orange color and ABTS to give a green color in the presence of H_2O_2. These results further verify the POD-mimicking activity of Fe_1@Au NPs.

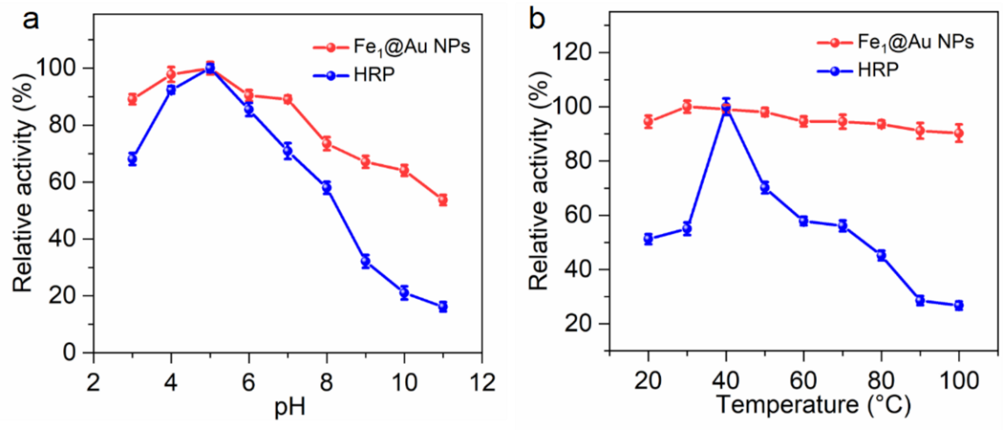

Supplementary Figure 13. The effect of different reaction conditions on peroxidase-like activity of Fe_1@Au NPs and HRP. (a) pH and (b) temperature. Error bars represent standard deviation from three independent measurements. Note: Experiment results reveal that the optimal pH and temperature of Fe_1@Au NPs are 5.0 and 30 °C in POD-mimicking catalytic reaction. Supplementary Fig. 13b shows variation in the catalytic activity of the Fe_1@Au NPs was less than 10% and HRP was more than 70% as the temperature changed from 20 to100 °C. These results indicate that the Fe_1@Au NPs possesses stable peroxidase-mimicking activity over a wide temperature range.

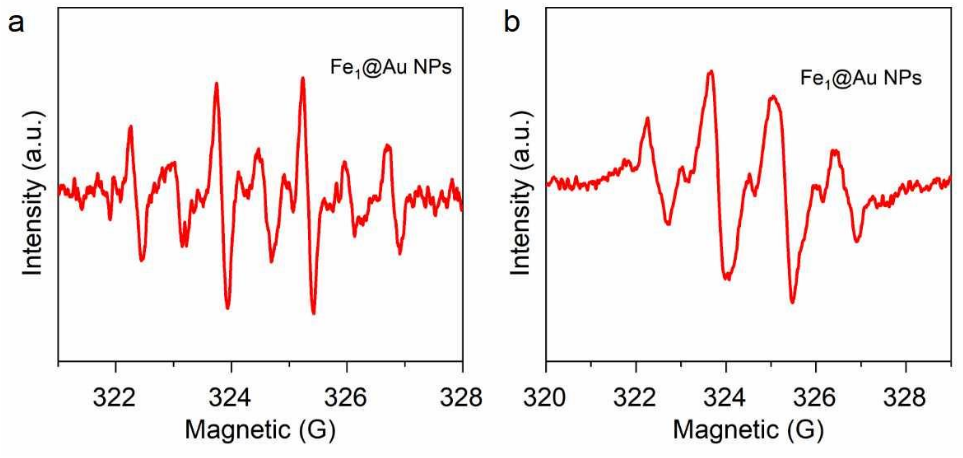

Supplementary Figure 14. EPR spectra of different reaction systems. (a) EPR spectrum of the DMPO-H adduct from the 5, 5-dimethyl-1-pyrroline 1-oxide (DMPO) and glucose reaction mixture in the presence of Fe_1@Au NPs. (b) EPR spectrum of Fe_1@Au NPs in the system containing H_2O_2 and DMPO.

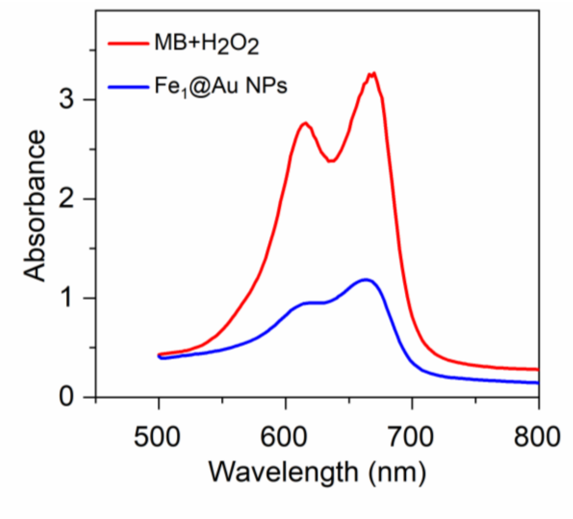

Supplementary Figure 15. Absorption spectra of Fe_1@Au NPs in H_2O_2/MB solution. Red line: the solution containing MB and H_2O_2. Blue line: the solution containing MB, H_2O_2 and Fe_1@Au NPs. Note: The degradation of methylene blue experiments verified the existence of hydroxyl radicals (·OH), and the degradation rates of methylene blue reflecting the relative content of ·OH are well consistent with their POD-mimicking activities.

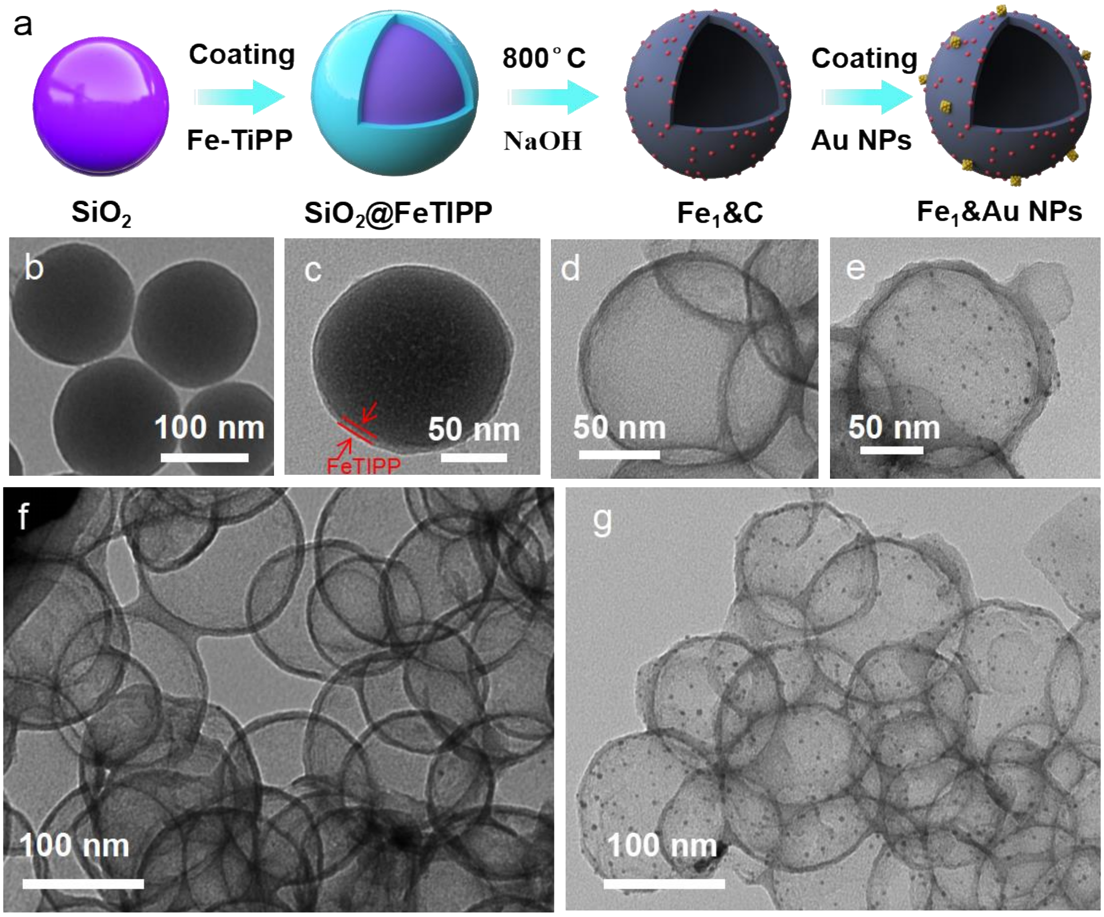

Supplementary Figure 16. Schematic illustrations and TEM images for the preparation of Fe_1&Au NPs and reference materials. (a) Scheme of the formation of Fe_1&Au NPs. TEM images of (b) SiO_2, (c) SiO_2@FeTIPP, (d, f) Fe_1&NC and (e, g) Fe_1&Au NPs. Note: Supplementary Fig. 16a presents the schematic for the fabrication the Fe_1&Au NPs. The SiO_2 was firstly synthesized. Then the obtained SiO_2 was dispersed in Fe-TIPP/TIPP solution as template before addition of another monomer. The obtained mixed solution performed quaterization. Subsequently, the collected product (Supplementary Fig. 16c) was annealed under flowing H_2/Ar gases and then etched with 5M NaOH to remove SiO_2 template.[8 9] Finally, the as-obtained Fe_1&NC was used as a template for growth of Au nanoparticles by reduction of $HAuCl_4$ with $NaBH_4$ to obtain the Fe_1&Au NPs (Supplementary Fig. 16e). TEM were used to record the evolution process. From Supplementary Fig. 16d-g, we can see that Fe_1&NC and Fe_1&Au NPs retains the SiO_2 shape, and its wall thickness is roughly 5 nm. In addition, no obviously nanoparticles or nanoclusters could be detected in Fe_1&NC (Supplementary Fig.16d, f).

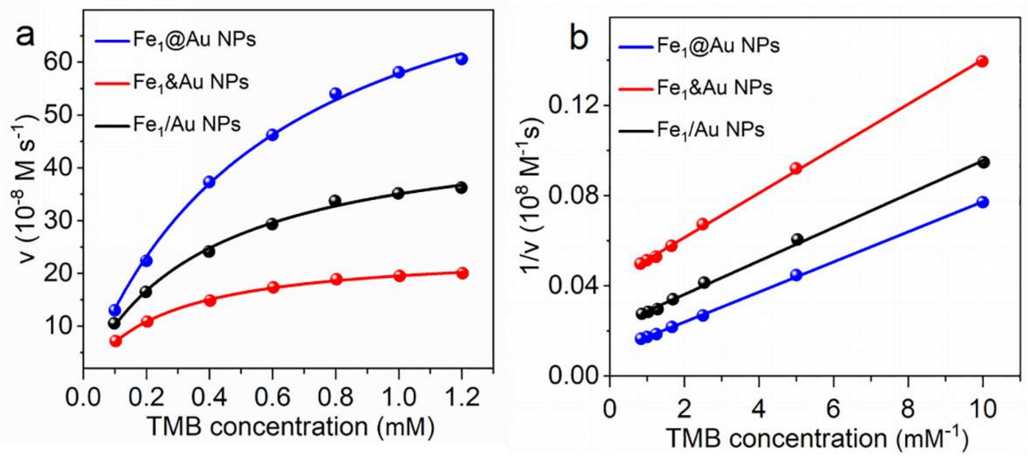

Supplementary Figure 17. Steady-state kinetic assay of POD-like activity. (a) Michaelis-Menten curves by varying TMB concentration at constant H_2O_2 concentration. (b) The corresponding Lineweaver-Burk plots with TMB as a substrate.

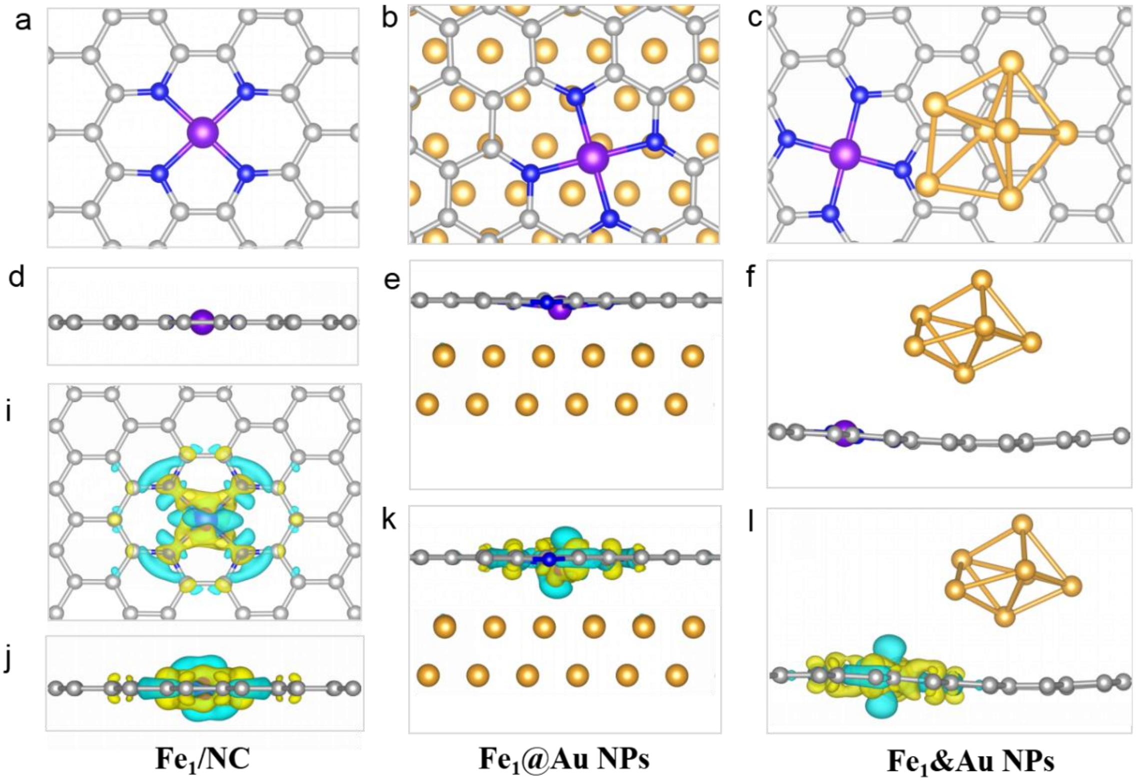

Supplementary Figure 18. DFT calculations of the POD-mimicking models on the optimized structure of Fe_1/NC, Fe_1@Au NPs and Fe_1&Au NPs. Fe_1/NC with (a) top and (d) side views, Fe_1@Au NPs with (b) top and (e) side views, and Fe_1&Au NPs with top (c) and side (f) views. Charge density difference from (i) top and (j) side views of Fe_1/NC, and the side views of (k) Fe_1@Au NPs and (l) Fe_1&Au NPs, where the isosurface value is set to 0.005 e/Å^3 and the positive and negative charges are shown in yellow and cyan, respectively. The gray, blue, and purple balls represent C, N, and Fe atoms, respectively. Note: For comparison, the catalytic behaviors of the Fe_1/NC catalyst are also calculated. DFT simulations of the Fe_1/NC show the similar shape of charge density difference with the Fe_1@Au NPs moiety (Fig. 3g), in which the charge accumulation depletion mainly occurred on the Fe-N bonds and Fe or C atoms. In addition, an apparent charge polarization is induced by modifying the charge distribution of the FeN_4 moiety by the Au NPs in Fe_1&Au NPs and Fe_1@Au NPs (Supplementary Fig. 18k-l).

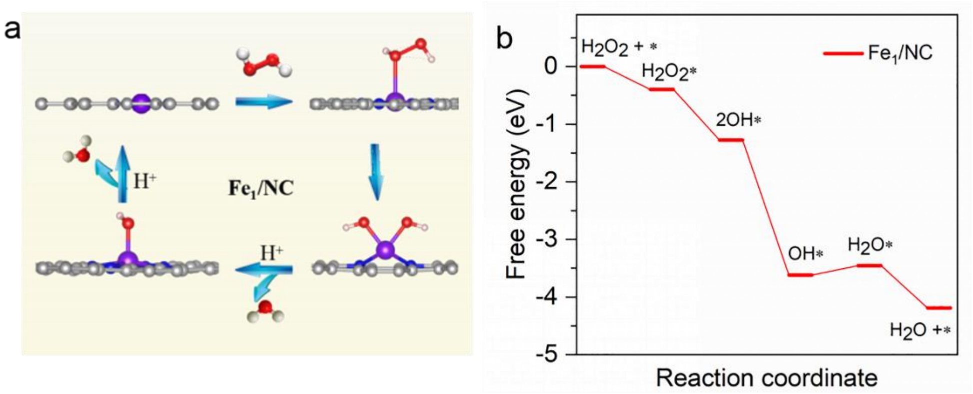

Supplementary Figure 19. The theoretical investigation of the mechanism for the POD-mimicking reaction of Fe_1/NC. (a) The catalytic mechanism along the POD-mimicking reaction path on the Fe_1/NC with side view. (b) The free-energy diagram for the POD-mimicking reaction on Fe_1/NC. The gray, blue, purple, red, and white balls represent C, N, Fe, O, and H atoms, respectively. Note: The corresponding free-energy profiles show the rate-determining step (RDS) of the POD-mimicking reaction for Fe_1/NC is the step of OH* protonation to H_2O* with energy barrier of +0.16 eV.

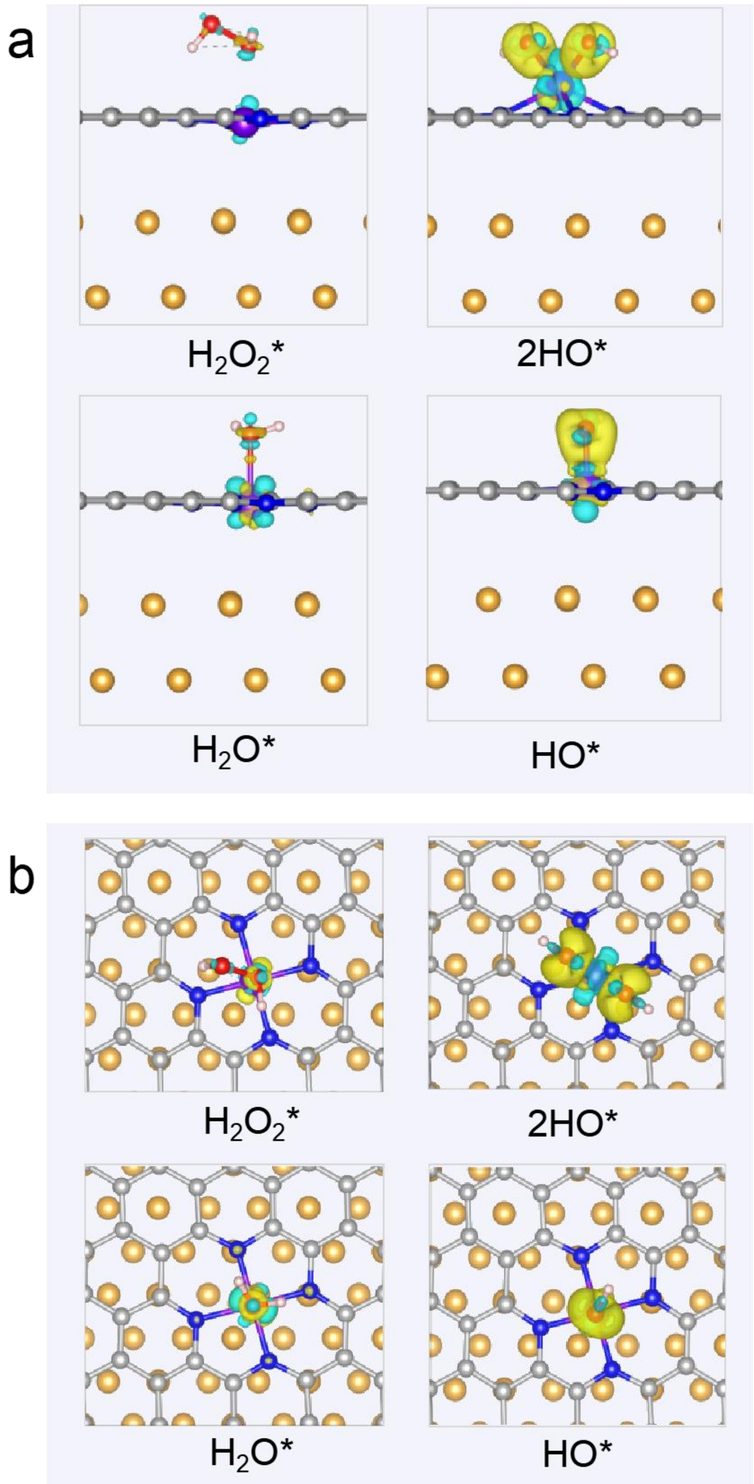

Supplementary Figure 20. Charge distribution analysis from DFT calculations. Charge density difference of Fe_1@Au NPs with (a) side and (b) top views after the H_2O_2, 2HO, HO and H_2O intermediates adsorption. The isosurface value is set to 0.005 e/Å^3 and the positive and negative charges are shown in yellow and cyan, respectively. The gray, blue, purple, light yellow, red, and white balls represent C, N, Fe, Au, O, and H atoms, respectively.

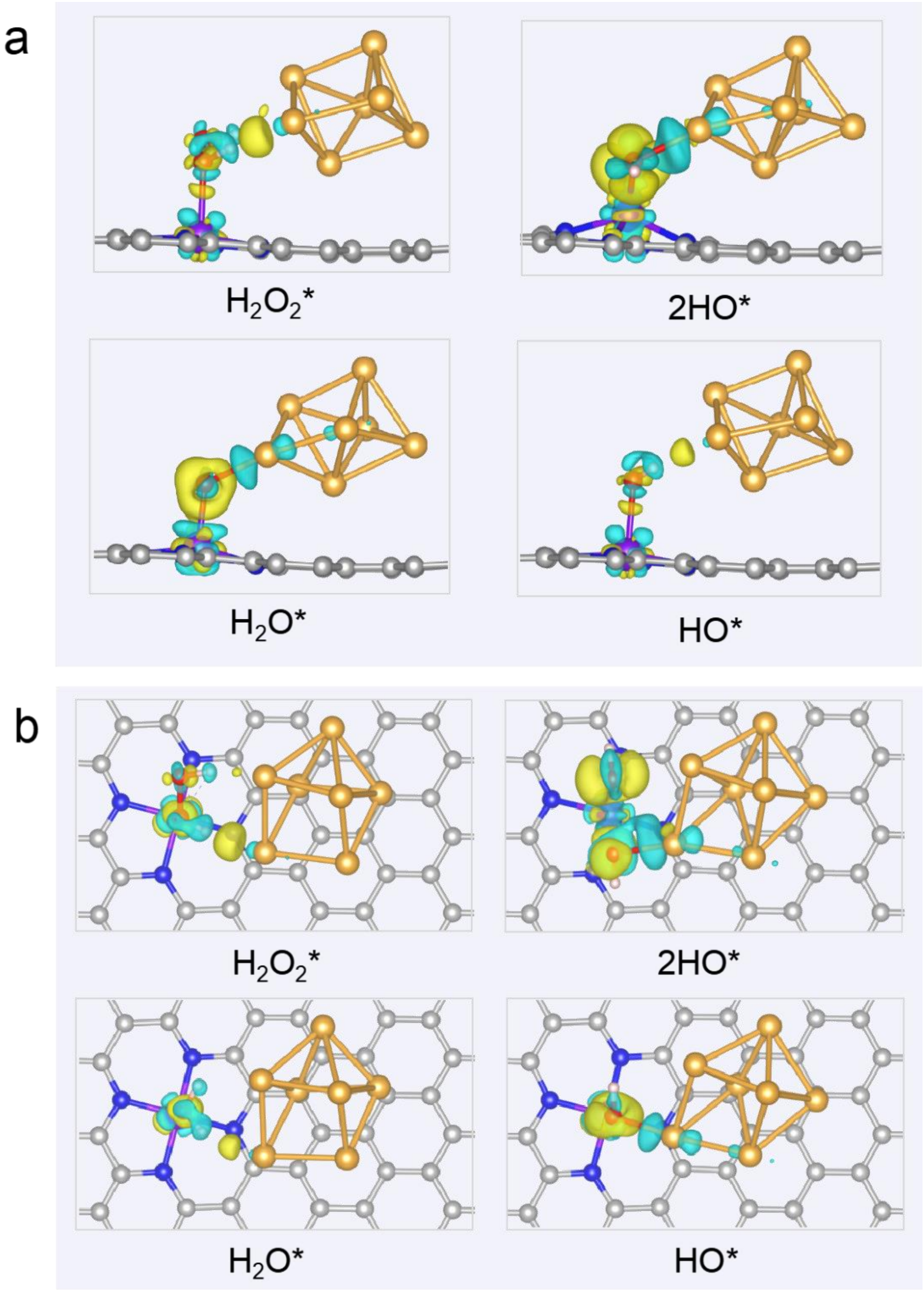

Supplementary Figure 21. Charge distribution analysis from DFT calculations. Charge density difference of Fe_1&Au NPs with (a) side and (b) top views after the H_2O_2, 2HO, HO and H_2O intermediates adsorption, where the isosurface value is set to 0.005 e/Å^3 and the positive and negative charges are shown in yellow and cyan, respectively. The gray, blue, purple, light yellow, red, and white balls represent C, N, Fe, Au, O, and H atoms, respectively.

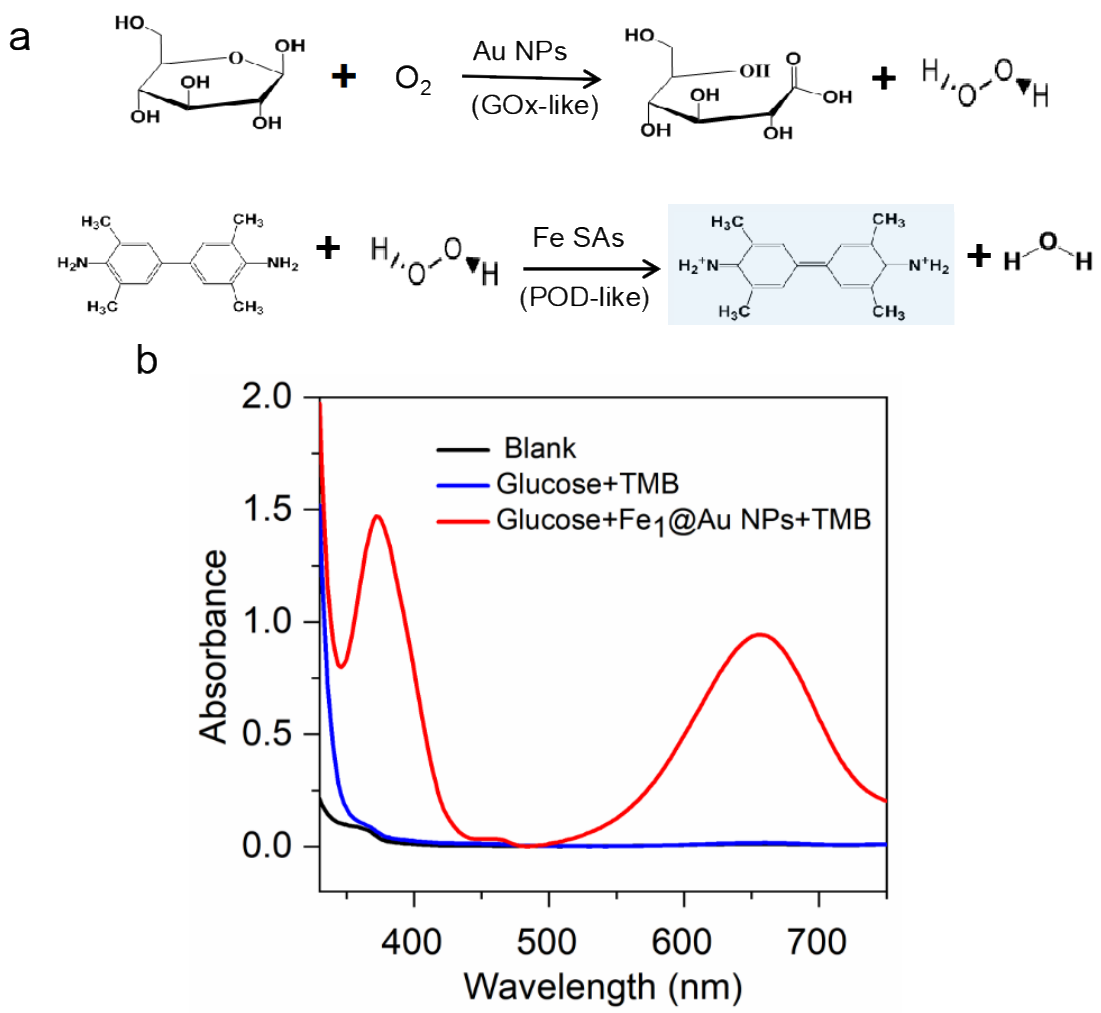

Supplementary Figure 22. The Fe_1@Au NPs-based system catalytic cascade reaction. (a) Schematic illustration of the biomimetic Fe_1@Au NPs system catalytic cascade reaction based on the colorimetric detection of glucose. (b) UV-Vis absorbance spectra at different assay samples.

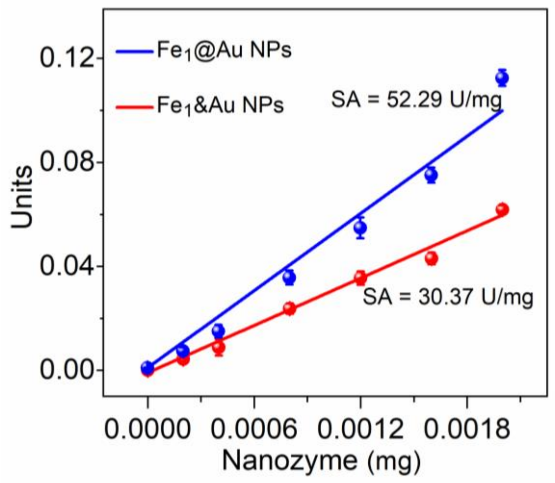

Supplementary Figure 23. Specific activities of Fe_1@Au NPs and Fe_1&Au NPs. Error bars represent standard deviation from three independent measurements.

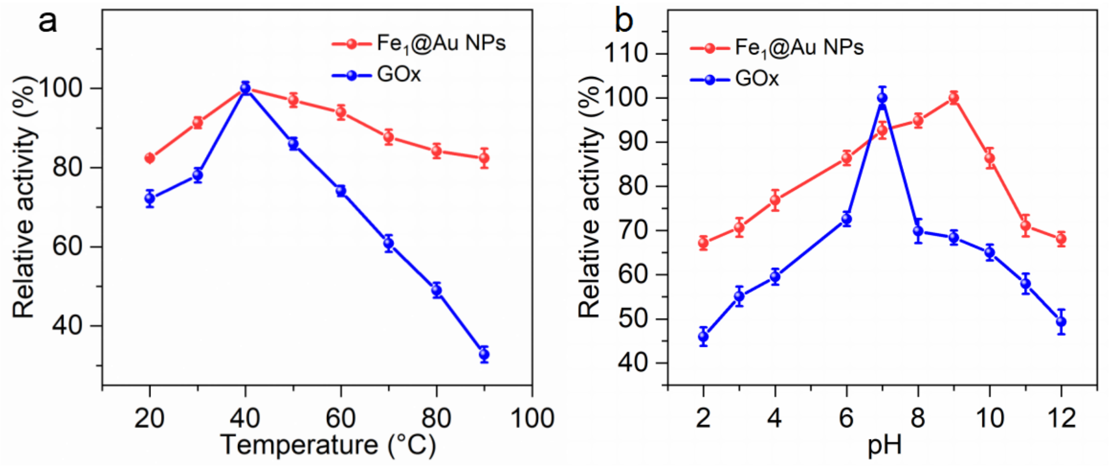

Supplementary Figure 24. The effect of different reaction conditions on GOx-like activity of Fe_1@Au NPs and GOx. (a) temperature, and (b) pH. Error bars represent standard deviation from three independent measurements.

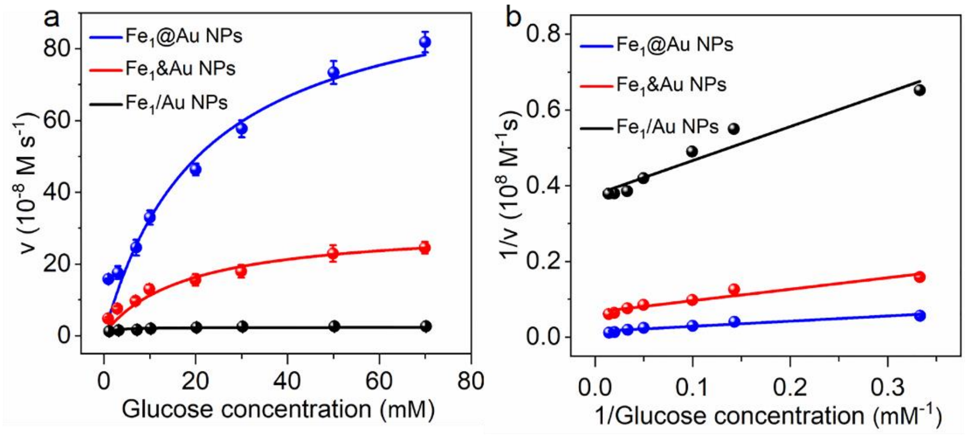

Supplementary Figure 25. Steady-state kinetic assay of GOx-like activity. (a) Michaelis-Menten curves by varying glucose concentration. (b) The corresponding Lineweaver-Burk plots with glucose as a substrate. Error bars represent standard deviation from three independent measurements.

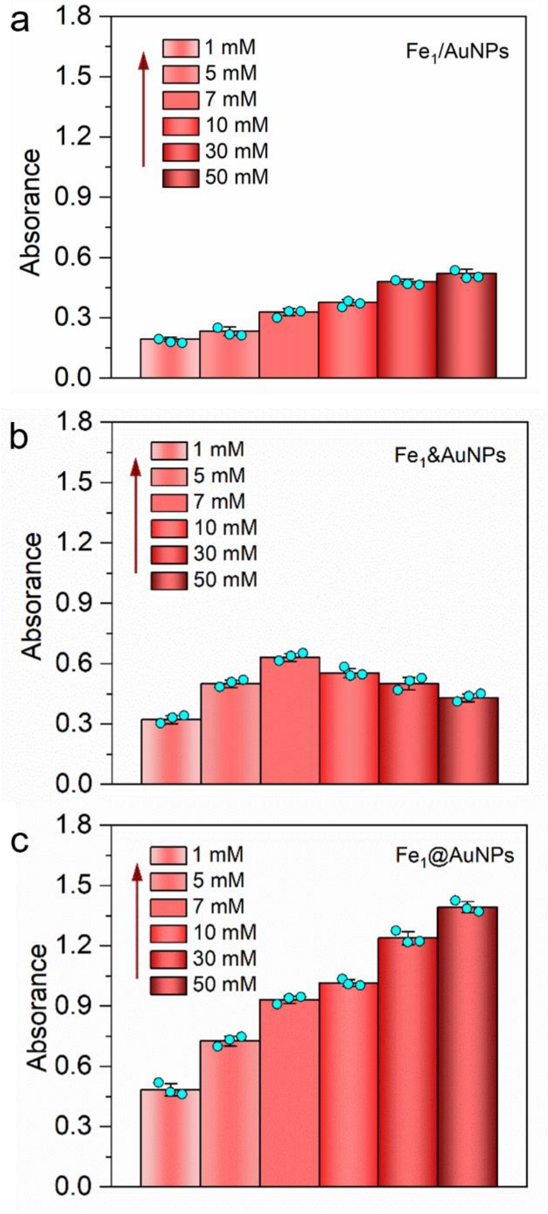

Supplementary Figure 26. The absorption changes of TMB at varying glucose concentrations. oxTMB generation by (a) Fe_1/Au NPs, (b) Fe_1&Au NPs, and (c) Fe_1@Au NPs after reacting with different glucose concentrations and 0.6 mM TMB for 1 h. Error bars represent standard deviation from three independent measurements.

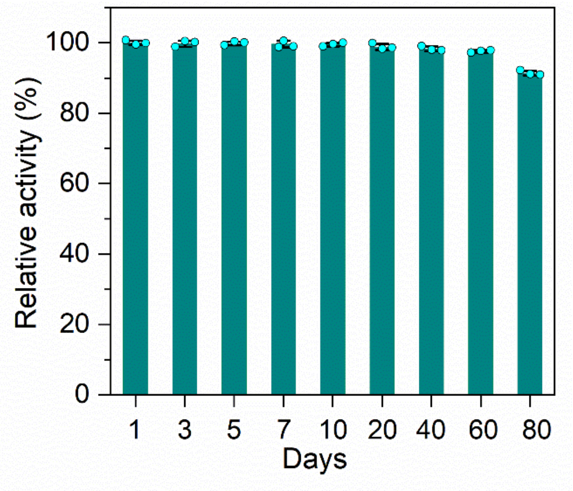

Supplementary Figure 27. **The storage stability of Fe_1@Au NPs-based colorimetric glucose biosensor.** Error bars represent standard deviation from three independent measurements. Note: The biomimetic Fe_1@Au NPs system exhibits no significant loss of bioactivity in long-term storage tests, indicating good long-term storage stability of Fe_1@Au NPs.

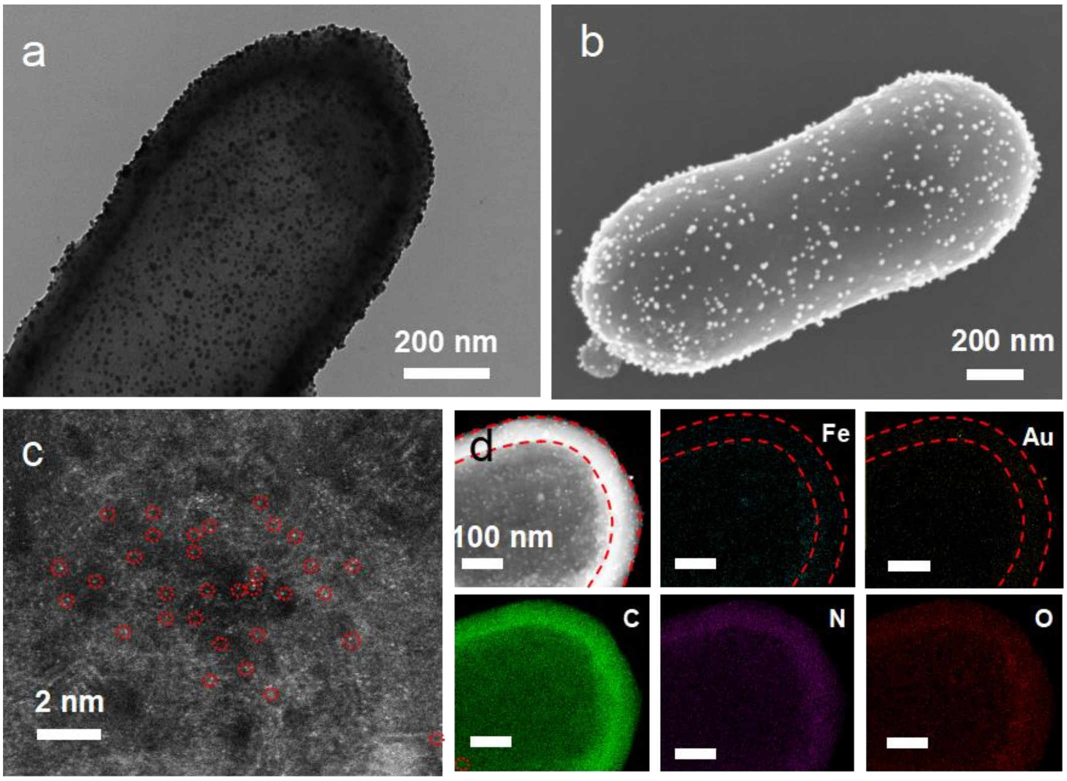

Supplementary Figure 28. **Morphology characterization of Fe_1@Au NPs after the catalytic recycling tests.** (a) TEM, (b) SEM, (c) AC HADDF-STEM, and (d) EDS elemental mapping images.

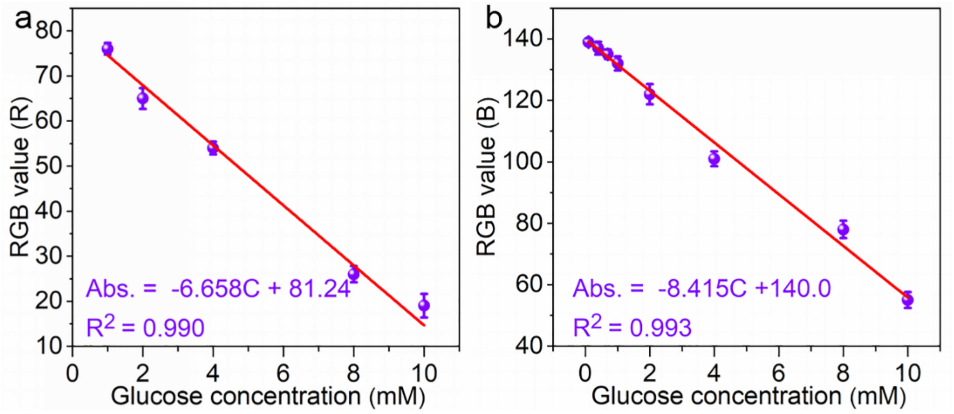

Supplementary Figure 29. Fe_1@Au NPs–based gel sensor for the colorimetric detection of glucose. Plots of the values of (a) R and (b) B versus the glucose concentration, respectively. Error bars represent standard deviation from three independent measurements. Note: To determine the most suitable relationship for the quantification of a scanned image, we analyzed different quantitative relationships, including G, R, and B. As shown in Fig. 5b and Supplementary Fig. 29, a good linear relationship was established between the RGB values and glucose concentrations in the range of 0.1–10 mM (R^2 = 0.998), 1–10 mM (R^2 = 0.990), and 0.4–10 mM (R^2 = 0.993) when G, R, and B were employed as the detection signal, respectively. Among all the quantitative relationships studied, G strongly correlated with the glucose concentration. Therefore, the intensity of G was selected as the analytical signal for detection.

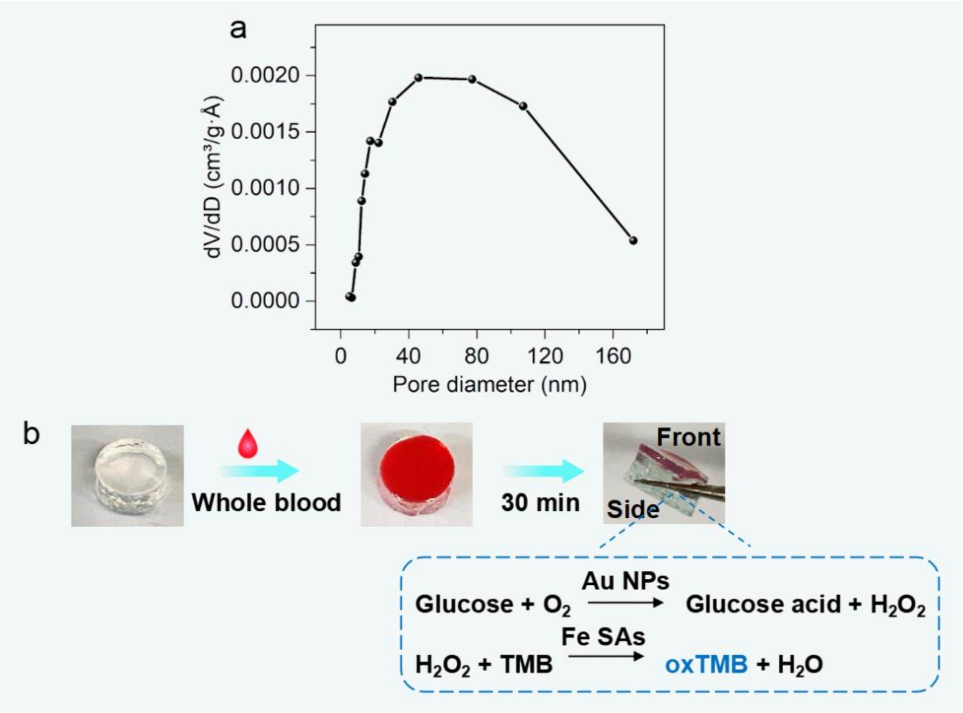

Supplementary Figure 30. **Characterizations of agarose-based gel and the detection of glucose in real samples.** (a) The pore-size distribution of agarose-based gel. (b) The detection of glucose in whole blood. Note: In our study, the obtained agarose-based gels (1% w/v) possess a porous structure with the size of nanometer scale (Supplementary Fig. 30a), which can allow small molecules (glucose) to pass through freely while preventing the entry of biological macromolecules (red blood cells, 7-8 μm diameter). When the whole blood sample was loaded into the prepared gel, small target molecules (glucose) pass through and were detected via a chromogenic reaction, while the red blood cells remained on the front of the gel. A smartphone was used to record the color signal at the side of the gel to achieve quantitative determination of the targets without any interference from the blood samples (Supplementary Fig. 30b).

Supplementary Table 1. The contents of Fe and Au in the different catalysts quantified by different methods.

**Catalysts**	**Fe_1@Au NPs**	**Fe_1&Au NPs**	**Fe_1/NC**
**ICP-MS (Fe, wt%)**	6.39	5.97	6.58
**ICP-MS (Au, wt%)**	2.08	1.96	-
**XPS (Fe, at%)**	0.4	1.2	0.4
**XPS (Au, at%)**	0.8	0.7	-

Supplementary Table 2. Parameters of the best Fe K-edge EXAFS Fitting results for the Fe_1@Au NPs catalyst.

**Catalysts**	**Path**	**CN**	**R(Å)**	**σ^2(10^{-3}Å2)**	**ΔE_0 (eV)**	**R-factor**
**Fe_1@Au NPs**	Fe-N	4.28	2.035	11.0	4.5	0.008

Supplementary Table 3. Comparison of the peroxidase-like kinetic parameters of natural enzyme and various nanozymes.

Catalysts	K_m (mM)		V_m (10^{-8} M/s)		Ref
	TMB	H_2O_2	TMB	H_2O_2	
Fe_1@Au NPs	0.598	1.83	92.37	166.33	This work
Fe_1&Au NPs	0.24	12.00	24.34	27.0	This work
Fe_1/Au NPs	0.38	2.9	48.51	2.58	This work
HRP	0.434	3.7	10	8.71	[10]
Fe_3O_4	0.098	154	3.44	9.78	[10]
AKCN	0.601	0.79	4.22	6.78	[11]
Au-PDA	1.53	89.52	1.18	2.01	[12]
Au hydrogel-HCl	2.25	38.67	3.56	3.98	[12]
Au hydrogel	0.32	19.92	12.30	12.8	[12]
EMSN-Au NPs	-	119.2	-	5.258	[13]

Supplementary Table 4. Comparison of the GOx-like kinetic parameters of natural enzyme and various nanozymes.

Catalysts	K_m (mM)	V_m (10^{-8} M/s)	Ref
Fe_1@Au NPs	2.90	113.5	This work
Fe_1&Au NPs	12.06	27.00	This work
Fe_1/Au NPs	1.83	2.58	This work
GOx	3.42	111.3	[14]
Au-PDA	3.39	5.56	[12]
Au hydrogel-HCl	7.91	33.6	[12]
Au hydrogel	4.98	82.1	[12]
EMSN-Au NPs	26.20	52.5	[13]
Au NPs	6.97	63	[14]

Supplementary Table 5. Bifunctional oxidase-peroxidase mimicking nanozymes operating in cascade catalysis for colorimetric glucose detection.

Catalysts	Regents/Probe	Linear range (μM)	LOD (μM)	Ref
Fe_1@Au NPs	TMB	0-1600	0.13	This work
Au NPs/Cu-TCPP	TMB	10-300	8.5	[4]
AKCN	TMB	-	0.8	[11]
Au/V_2O_5	ABTS	0-10	0.5	[15]
Au_1/CeO_2	TMB	10-100	10	[16]
Au@Pt	OPD	45-400	45	[17]
MnO_2	TMB	5-1200	3.3	[18]
Au NPs-Ag NPs	-	5-70	3	[19]
Ag-Au NC-30@CeO_2	TMB	20-240	20	[20]
Au@BSA-GO	TMB	10-300	0.6	[21]
AuPd-NE aerogels	TMB	30-250	10	[22]

Supplementary Methods

DFT calculations. The spin-polarized periodic density functional theory (DFT) calculations were performed via Vienna *ab initio* simulation package (VASP.5.4.4).[23,24] The ion-electron interaction was described with the projector-augmented plane-wave (PAW) method.[2] Exchange-correlation energy were expressed by Perdew-Burke-Ernzerhof (PBE) functional with the generalized gradient approximation (GGA).[25] In this study, the Fe SAs was built with the FeN_4 moiety decorated graphene layer. The Fe_1@AuNPs catalyst was constructed with stacking the Fe SAs layer on top of the Au (111) surface. While the Fe_1&AuNPs was built with a small Au cluster containing seven Au atoms neighboring the FeN_4 moiety of the Fe SAs layer. To avoid the interlayer interaction the vacuum layer of these structures were set to be 15 Å. For geometry optimization, the cut-off energy was set to be 520 eV and the Brillouin zone was sampled with 3×3×1 k-points. The systems were relaxed until the energy and force reaching the convergence threshold of 10^{-5} eV and 0.02 eV/Å, respectively. We describe the van der Waals (vdW) interactions by utilizing the DFT-D3 method.[26] The electronic structures of the density of state (DOS) were calculated with 7×7×1 k-points.

Generally, the peroxidase-like reaction mechanism along with the following five elementary steps were considered for these peroxidase-like nanozymes.

$$H_2O_2 + * \rightarrow H_2O_2* \quad \text{(S1)}$$

$$H_2O_2* \rightarrow 2HO* \quad \text{(S2)}$$

$$2HO* \rightarrow HO* + \cdot OH \quad \text{(S3)}$$

$$HO* + H^+ + e^- \rightarrow H_2O* \quad \text{(S4)}$$

$$H_2O* \rightarrow H_2O + * \quad \text{(S5)}$$

Where the asterisk represents the adsorption site. The Gibbs free energy change ΔG on Fe_1&Au NPs, Fe_1/Au NPs, and Fe_1@Au NPs were evaluated by the formula:

$$\Delta G = \Delta E + \Delta ZPE + \Delta \int_0^T C_p dT - T\Delta S + \Delta G_U + \Delta G_{pH} \quad \text{(S6)}$$

Where ΔE is the adsorption energy of adsorbates and ΔZPE is their corresponding zero-point energy. ΔG_U is the free energy contribution induced by electrode potential U. ΔG_{pH} is the correction of the H^+ free energy by the concentration, which can be evaluated as $\Delta G_{pH} = 2.303 \times k_B T \times pH$ (or 0.06 × pH). Here the value of pH was assumed to be zero. C_P is the constant-pressure heat capacity, the entropy and the integration terms are calculated based on the vibrational energies of adsorbates.

Supplementary References

1. Li, D. *et al.* A Defect-Driven Metal-free Electrocatalyst for Oxygen Reduction in Acidic Electrolyte. *Chem* **4**, 2345-2356 (2018).
2. Emmanuel T., R. & Photini, P. Reactivity of 6-phosphogluconolactone with hydroxylamine: The possible involvement of glucose-6-phosphate dehydrogenase in endogenous glycation reactions. *Chem. Biol. Interact.* **113**, 205-216 (1998).
3. Weijie, L. *et al.* Self-Catalyzed, Self-Limiting Growth of Glucose Oxidase-Mimicking Gold Nanoparticles. *ACS Nano* **4**, 7451-7458 (2010).
4. Huang, Y. *et al.* Growth of Au Nanoparticles on 2D Metalloporphyrinic Metal-Organic Framework Nanosheets Used as Biomimetic Catalysts for Cascade Reactions. *Adv. Mater.* **29,** 1700102 (2017).
5. Kiyoshi, Y., Terumichi, N. & Toyozo, U. Statistical Moments in Pharmacokinetics. *J. Pharmacokinet. Biop.* **6**, 6 (1977).
6. P. David, J., Thomas, E. & Ronald P., M. The Horseradish Peroxidase-catalyzed Oxidation of 3,5,3',5'-Tetramethylbenzidine. *J. Biol. Chem.* **257**, 3669-3675 (1982).
7. Jiang, B. *et al.* Standardized assays for determining the catalytic activity and kinetics of peroxidase-like nanozymes. *Nat. Protoc.* **13**, 1506-1520 (2018).
8. Han, Y.H. *et al.* Hollow N-Doped Carbon Spheres with Isolated Cobalt Single Atomic Sites: Superior Electrocatalysts for Oxygen Reduction. *J. Am. Chem. Soc.* **139**, 17269-17272 (2017).
9. Zhao, Y.F. *et al.* Simultaneous oxidative and reductive reactions in one system by atomic design. *Nat.Catal.* **4**, 134-143 (2021).
10. Gao, L. *et al.* Intrinsic peroxidase-like activity of ferromagnetic nanoparticles. *Nat. Nanotechnol.* **2**, 577-583 (2007).
11. Zhang, P. *et al.* Modified carbon nitride nanozyme as bifunctional glucose oxidase-peroxidase for metal-free bioinspired cascade photocatalysis. *Nat. Commun.* **10**, 940 (2019).
12. Jiao, L. *et al.* A dopamine-induced Au hydrogel nanozyme for enhanced biomimetic catalysis. *Chem. Commun.* **55**, 9865-9868 (2019).
13. Lin, Y.H., Li, Z.H., Chen, Z.W., Ren, J.S. & Qu, X.G. Mesoporous silica-encapsulated gold nanoparticles as artificial enzymes for self-activated cascade catalysis. *Biomaterials* **34**, 2600-2610 (2013).
14. Luo, W. *et al.* Self-Catalyzed, Self-Limiting Growth of Glucose Oxidase-Mimicking Gold Nanoparticles. *ACS Nano* **4**, 7451-7458 (2010).
15. Qu, K.G., Shi, P., Ren, J.S. & Qu, X.G. Nanocomposite Incorporating V_2O_5 Nanowires and Gold Nanoparticles for Mimicking an Enzyme Cascade Reaction and Its Application in the Detection of Biomolecules. *Chem. Eur. J.* **20**, 7501-7506 (2014).
16. Chen, M. *et al.* Facet Engineering of Nanoceria for Enzyme-Mimetic Catalysis. *ACS Appl. Mater. Interfaces* **14**, 21989–21995 (2022).
17. Liu, J. *et al.* Au@Pt core/shell nanorods with peroxidase- and ascorbate oxidase-like activities for improved detection of glucose. *Sens. Actuators B Chem.* **166-167**, 708-714 (2012).
18. Han, L., Zhang, H., Chen, D. & Li, F. Protein-Directed Metal Oxide Nanoflakes with Tandem Enzyme-Like Characteristics: Colorimetric Glucose Sensing Based on One-

Pot Enzyme-Free Cascade Catalysis. *Adv. Funct. Mater.* **28,** 1800018 (2018).

19. Gao, Y., Wu, Y. & Di, J. Colorimetric detection of glucose based on gold nanoparticles coupled with silver nanoparticles. *Spectrochim. Acta A Mol. Biomol. Spectrosc.* **173**, 207-212 (2017).
20. Zhang, L. *et al.* CeO_2-Encapsulated Hollow Ag-Au Nanocage Hybrid Nanostructures as High-Performance Catalysts for Cascade Reactions. *Small* **15**, e1903182 (2019).
21. Zhang, H., Liang, X., Han, L. & Li, F. "Non-Naked" Gold with Glucose Oxidase-Like Activity: A Nanozyme for Tandem Catalysis. *Small* **14**, e1803256 (2018).
22. Xu, R. *et al.* Norepinephrine-induced AuPd aerogels with peroxidase- and glucose oxidase-like activity for colorimetric determination of glucose. *Mikrochim Acta* **188**, 362 (2021).
23. Kresse, G. Ab initio molecular dynamics for liquid metals. *J. Non Cryst. Solids* **192**, 222-229 (1995).
24. Kresse, G. & Hafner, J. Ab initio molecular dynamics for liquid metals. *Phys. Rev. B Condens. Matter* **47**, 558-561 (1993).
25. Perdew, J.P., Burke, K. & Ernzerhof, M. Generalized Gradient Approximation Made Simple. *Phys. Rev. Lett.* **77**, 3865-3868 (1996).
26. Grimme, S. Semiempirical GGA-type density functional constructed with a long-range dispersion correction. *J. Comput. Chem.* **27**, 1787-1799 (2006).
