# Supplementary material for: Cell-inspired design of cascade catalysis system by 3D spatially separated active sites
Source: Nat Commun. 2023 Sep 2;14:5338. doi: 10.1038/s41467-023-41002-5 (PMC10475024; doi:10.1038/s41467-023-41002-5)
Supplement: Supplementary file 3 — Description of Additional Supplementary files [file 41467_2023_41002_MOESM3_ESM.pdf]

Description of Additional Supplementary files

File name: Supplementary Data 1

Description: Optimized model of pure catalyst and configuration of H_2O/H_2O_2 adsorption catalysts.
