# Supplementary material for: Cell-inspired design of cascade catalysis system by 3D spatially separated active sites
Source: Nat Commun. 2023 Sep 2;14:5338. doi: 10.1038/s41467-023-41002-5 (PMC10475024; doi:10.1038/s41467-023-41002-5)
Supplement: Supplementary file 4 — Supplementary Data 1 [file 41467_2023_41002_MOESM4_ESM.pdf]

```
Fe1@Au NPs

#=====================================================================
=
# CRYSTAL DATA
#---------------------------------------------------------------------
-
data_VESTA_phase_1

_chemical_name_common                  ' Fe1@Au NPs'
_cell_length_a                         10.028700
_cell_length_b                         8.685200
_cell_length_c                         25.584900
_cell_angle_alpha                      90.000000
_cell_angle_beta                       90.000000
_cell_angle_gamma                      90.000000
_cell_volume                           2228.477057
_space_group_name_H-M_alt              'P 1'
_space_group_IT_number                 1

loop_
_space_group_symop_operation_xyz
   'x, y, z'

loop_
   _atom_site_label
   _atom_site_occupancy
   _atom_site_fract_x
   _atom_site_fract_y
   _atom_site_fract_z
   _atom_site_adp_type
   _atom_site_U_iso_or_equiv
   _atom_site_type_symbol
   Au1         1.0     0.000000     0.000000     0.078170    Uiso   ? Au
   Au2         1.0     0.000064    -0.000019     0.359924    Uiso   ? Au
   Au3         1.0     0.000000     0.333330     0.078170    Uiso   ? Au
   Au4         1.0    -0.000105     0.333436     0.359810    Uiso   ? Au
   Au5         1.0     0.000000     0.666670     0.078170    Uiso   ? Au
   Au6         1.0    -0.000168     0.666565     0.359867    Uiso   ? Au
   Au7         1.0     0.333357     0.000038     0.265006    Uiso   ? Au
   Au8         1.0     0.334423     0.333330     0.265493    Uiso   ? Au
   Au9         1.0     0.334336     0.666564     0.265448    Uiso   ? Au
   Au10        1.0     0.166670     0.000000     0.172300    Uiso   ? Au
   Au11        1.0     0.166670     0.333330     0.172300    Uiso   ? Au
```

Au12	1.0	0.166670	0.666670	0.172300	Uiso	?	Au
Au13	1.0	0.083371	0.166714	0.265022	Uiso	?	Au
Au14	1.0	0.084597	0.500024	0.264780	Uiso	?	Au
Au15	1.0	0.083356	0.833317	0.265021	Uiso	?	Au
Au16	1.0	0.416670	0.166670	0.172300	Uiso	?	Au
Au17	1.0	0.416670	0.500000	0.172300	Uiso	?	Au
Au18	1.0	0.416670	0.833340	0.172300	Uiso	?	Au
Au19	1.0	0.250000	0.166670	0.078170	Uiso	?	Au
Au20	1.0	0.250111	0.166663	0.359930	Uiso	?	Au
Au21	1.0	0.250000	0.500000	0.078170	Uiso	?	Au
Au22	1.0	0.249964	0.500018	0.359929	Uiso	?	Au
Au23	1.0	0.250000	0.833340	0.078170	Uiso	?	Au
Au24	1.0	0.250048	0.833297	0.359886	Uiso	?	Au
Au25	1.0	0.500000	0.000000	0.078170	Uiso	?	Au
Au26	1.0	0.499977	-0.000129	0.359951	Uiso	?	Au
Au27	1.0	0.500000	0.333330	0.078170	Uiso	?	Au
Au28	1.0	0.500620	0.334049	0.362576	Uiso	?	Au
Au29	1.0	0.500000	0.666670	0.078170	Uiso	?	Au
Au30	1.0	0.500368	0.665225	0.361999	Uiso	?	Au
Au31	1.0	0.833275	0.000151	0.264978	Uiso	?	Au
Au32	1.0	0.833206	0.334135	0.265235	Uiso	?	Au
Au33	1.0	0.833066	0.666237	0.265447	Uiso	?	Au
Au34	1.0	0.666670	0.000000	0.172300	Uiso	?	Au
Au35	1.0	0.666670	0.333330	0.172300	Uiso	?	Au
Au36	1.0	0.666670	0.666670	0.172300	Uiso	?	Au
Au37	1.0	0.583073	0.167091	0.265459	Uiso	?	Au
Au38	1.0	0.583716	0.499635	0.265517	Uiso	?	Au
Au39	1.0	0.583116	0.832904	0.265437	Uiso	?	Au
Au40	1.0	0.916670	0.166670	0.172300	Uiso	?	Au
Au41	1.0	0.916670	0.500000	0.172300	Uiso	?	Au
Au42	1.0	0.916670	0.833340	0.172300	Uiso	?	Au
Au43	1.0	0.750000	0.166670	0.078170	Uiso	?	Au
Au44	1.0	0.750097	0.166722	0.359929	Uiso	?	Au
Au45	1.0	0.750000	0.500000	0.078170	Uiso	?	Au
Au46	1.0	0.749127	0.499811	0.362215	Uiso	?	Au
Au47	1.0	0.750000	0.833340	0.078170	Uiso	?	Au
Au48	1.0	0.750006	0.833372	0.359933	Uiso	?	Au
C1	1.0	-0.000219	0.002428	0.493467	Uiso	?	C
C2	1.0	0.121415	0.248476	0.493257	Uiso	?	C
C3	1.0	-0.005249	0.170196	0.493048	Uiso	?	C
C4	1.0	0.125390	0.414584	0.492844	Uiso	?	C
C5	1.0	-0.000013	0.502196	0.492848	Uiso	?	C
C6	1.0	0.130761	0.746736	0.492993	Uiso	?	C
C7	1.0	0.004021	0.668434	0.493094	Uiso	?	C

C8	1.0	0.125763	0.914426	0.493603	Uiso	? C
C9	1.0	0.250111	-0.005276	0.493872	Uiso	? C
C10	1.0	0.369490	0.243245	0.492200	Uiso	? C
C11	1.0	0.246527	0.161107	0.493496	Uiso	? C
C12	1.0	0.254975	0.491124	0.491276	Uiso	? C
C13	1.0	0.384672	0.744900	0.491715	Uiso	? C
C14	1.0	0.258655	0.659390	0.491677	Uiso	? C
C15	1.0	0.376259	0.911205	0.493545	Uiso	? C
C16	1.0	0.498921	-0.002671	0.493428	Uiso	? C
C17	1.0	0.496528	0.160046	0.492283	Uiso	? C
C18	1.0	0.629124	0.756895	0.492325	Uiso	? C
C19	1.0	0.626628	0.919532	0.493602	Uiso	? C
C20	1.0	0.749182	0.005566	0.493608	Uiso	? C
C21	1.0	0.866864	0.257545	0.491781	Uiso	? C
C22	1.0	0.740771	0.172026	0.491638	Uiso	? C
C23	1.0	0.870547	0.425671	0.491504	Uiso	? C
C24	1.0	0.878932	0.755789	0.493671	Uiso	? C
C25	1.0	0.755984	0.673785	0.492251	Uiso	? C
C26	1.0	0.875331	0.922062	0.493903	Uiso	? C
N1	1.0	0.373434	0.402563	0.489700	Uiso	? N
N2	1.0	0.614723	0.244633	0.489856	Uiso	? N
N3	1.0	0.510800	0.672245	0.489555	Uiso	? N
N4	1.0	0.752005	0.514293	0.489481	Uiso	? N
Fe1	1.0	0.562711	0.458270	0.481197	Uiso	? Fe

H2O-Fe1@Au NPs

```
#======================================================================
=
# CRYSTAL DATA
#----------------------------------------------------------------------
-
data_VESTA_phase_1

_chemical_name_common                  'H2O-Fe1@Au NPs'
_cell_length_a                         10.028700
_cell_length_b                         8.685200
_cell_length_c                         25.584900
_cell_angle_alpha                      90.000000
_cell_angle_beta                       90.000000
_cell_angle_gamma                      90.000000
_cell_volume                           2228.477057
_space_group_name_H-M_alt              'P 1'
_space_group_IT_number                 1
```

```
loop_
_space_group_symop_operation_xyz
   'x, y, z'

loop_
   _atom_site_label
   _atom_site_occupancy
   _atom_site_fract_x
   _atom_site_fract_y
   _atom_site_fract_z
   _atom_site_adp_type
   _atom_site_U_iso_or_equiv
   _atom_site_type_symbol
   Au1        1.0     0.000000    0.000000    0.078170    Uiso   ? Au
   Au2        1.0     0.000142   -0.000021    0.359855    Uiso   ? Au
   Au3        1.0     0.000000    0.333330    0.078170    Uiso   ? Au
   Au4        1.0     0.999765    0.333489    0.359701    Uiso   ? Au
   Au5        1.0     0.000000    0.666670    0.078170    Uiso   ? Au
   Au6        1.0     0.999719    0.666460    0.359788    Uiso   ? Au
   Au7        1.0     0.333389    0.000198    0.264978    Uiso   ? Au
   Au8        1.0     0.334616    0.333301    0.265374    Uiso   ? Au
   Au9        1.0     0.334576    0.666697    0.265294    Uiso   ? Au
   Au10       1.0     0.166670    0.000000    0.172300    Uiso   ? Au
   Au11       1.0     0.166670    0.333330    0.172300    Uiso   ? Au
   Au12       1.0     0.166670    0.666670    0.172300    Uiso   ? Au
   Au13       1.0     0.083421    0.166894    0.264998    Uiso   ? Au
   Au14       1.0     0.085102    0.500224    0.264623    Uiso   ? Au
   Au15       1.0     0.083376    0.833331    0.265015    Uiso   ? Au
   Au16       1.0     0.416670    0.166670    0.172300    Uiso   ? Au
   Au17       1.0     0.416670    0.500000    0.172300    Uiso   ? Au
   Au18       1.0     0.416670    0.833340    0.172300    Uiso   ? Au
   Au19       1.0     0.250000    0.166670    0.078170    Uiso   ? Au
   Au20       1.0     0.250199    0.166714    0.359876    Uiso   ? Au
   Au21       1.0     0.250000    0.500000    0.078170    Uiso   ? Au
   Au22       1.0     0.249970    0.500020    0.359874    Uiso   ? Au
   Au23       1.0     0.250000    0.833340    0.078170    Uiso   ? Au
   Au24       1.0     0.250103    0.833267    0.359802    Uiso   ? Au
   Au25       1.0     0.500000    0.000000    0.078170    Uiso   ? Au
   Au26       1.0     0.499983   -0.000008    0.359886    Uiso   ? Au
   Au27       1.0     0.500000    0.333330    0.078170    Uiso   ? Au
   Au28       1.0     0.500576    0.334386    0.362524    Uiso   ? Au
   Au29       1.0     0.500000    0.666670    0.078170    Uiso   ? Au
   Au30       1.0     0.500024    0.666059    0.362162    Uiso   ? Au
```

Au31	1.0	0.833288	0.000137	0.264979	Uiso	? Au
Au32	1.0	0.833219	0.334130	0.265197	Uiso	? Au
Au33	1.0	0.833186	0.666082	0.265371	Uiso	? Au
Au34	1.0	0.666670	0.000000	0.172300	Uiso	? Au
Au35	1.0	0.666670	0.333330	0.172300	Uiso	? Au
Au36	1.0	0.666670	0.666670	0.172300	Uiso	? Au
Au37	1.0	0.583369	0.167443	0.265468	Uiso	? Au
Au38	1.0	0.583208	0.500037	0.265436	Uiso	? Au
Au39	1.0	0.583162	0.833088	0.265426	Uiso	? Au
Au40	1.0	0.916670	0.166670	0.172300	Uiso	? Au
Au41	1.0	0.916670	0.500000	0.172300	Uiso	? Au
Au42	1.0	0.916670	0.833340	0.172300	Uiso	? Au
Au43	1.0	0.750000	0.166670	0.078170	Uiso	? Au
Au44	1.0	0.750134	0.166683	0.359872	Uiso	? Au
Au45	1.0	0.750000	0.500000	0.078170	Uiso	? Au
Au46	1.0	0.747852	0.499661	0.361383	Uiso	? Au
Au47	1.0	0.750000	0.833340	0.078170	Uiso	? Au
Au48	1.0	0.750020	0.833348	0.359850	Uiso	? Au
C1	1.0	0.002476	0.003921	0.492170	Uiso	? C
C2	1.0	0.123971	0.249946	0.491883	Uiso	? C
C3	1.0	0.997178	0.171753	0.491829	Uiso	? C
C4	1.0	0.128037	0.415923	0.491384	Uiso	? C
C5	1.0	0.002866	0.503726	0.491300	Uiso	? C
C6	1.0	0.133632	0.748085	0.491815	Uiso	? C
C7	1.0	0.006783	0.669883	0.491513	Uiso	? C
C8	1.0	0.128413	0.915851	0.492381	Uiso	? C
C9	1.0	0.252665	0.996235	0.492460	Uiso	? C
C10	1.0	0.371837	0.244784	0.490701	Uiso	? C
C11	1.0	0.248977	0.162609	0.491912	Uiso	? C
C12	1.0	0.257686	0.492095	0.490195	Uiso	? C
C13	1.0	0.387325	0.746723	0.490655	Uiso	? C
C14	1.0	0.261321	0.660852	0.490838	Uiso	? C
C15	1.0	0.378765	0.912944	0.491968	Uiso	? C
C16	1.0	0.501532	0.998784	0.491521	Uiso	? C
C17	1.0	0.499336	0.161380	0.490618	Uiso	? C
C18	1.0	0.631715	0.758577	0.490473	Uiso	? C
C19	1.0	0.629345	0.921209	0.491528	Uiso	? C
C20	1.0	0.751930	0.006936	0.491669	Uiso	? C
C21	1.0	0.869449	0.259003	0.490690	Uiso	? C
C22	1.0	0.743043	0.173314	0.490226	Uiso	? C
C23	1.0	0.873444	0.427353	0.490304	Uiso	? C
C24	1.0	0.881769	0.757396	0.491750	Uiso	? C
C25	1.0	0.758920	0.675368	0.490429	Uiso	? C
C26	1.0	0.878072	0.923579	0.492180	Uiso	? C

```
   N1          1.0     0.375755     0.403720     0.488938     Uiso  ? N
   N2          1.0     0.617275     0.245421     0.488771     Uiso  ? N
   N3          1.0     0.513337     0.674192     0.488664     Uiso  ? N
   N4          1.0     0.755094     0.515823     0.488618     Uiso  ? N
   Fe1         1.0     0.565321     0.459611     0.487368     Uiso  ? Fe
   H1          1.0     0.518283     0.550288     0.585782     Uiso  ? H
   H2          1.0     0.657355     0.471505     0.585560     Uiso  ? H
   O1          1.0     0.564913     0.457236     0.574700     Uiso  ? O
```

H2O2-Fe1@Au NPs

```
#======================================================================
=
# CRYSTAL DATA
#----------------------------------------------------------------------
-
data_VESTA_phase_1

_chemical_name_common                  ' H2O2-Fe1@Au NPs'
_cell_length_a                         10.028700
_cell_length_b                         8.685200
_cell_length_c                         25.584900
_cell_angle_alpha                      90.000000
_cell_angle_beta                       90.000000
_cell_angle_gamma                      90.000000
_cell_volume                           2228.477057
_space_group_name_H-M_alt              'P 1'
_space_group_IT_number                 1

loop_
_space_group_symop_operation_xyz
   'x, y, z'

loop_
   _atom_site_label
   _atom_site_occupancy
   _atom_site_fract_x
   _atom_site_fract_y
   _atom_site_fract_z
   _atom_site_adp_type
   _atom_site_U_iso_or_equiv
   _atom_site_type_symbol
   Au1         1.0     0.000000     0.000000     0.078170     Uiso  ? Au
   Au2         1.0     0.000063    -0.000015     0.359912     Uiso  ? Au
```

Au3	1.0	0.000000	0.333330	0.078170	Uiso	? Au
Au4	1.0	0.999872	0.333470	0.359806	Uiso	? Au
Au5	1.0	0.000000	0.666670	0.078170	Uiso	? Au
Au6	1.0	0.999813	0.666516	0.359864	Uiso	? Au
Au7	1.0	0.333396	0.000160	0.265000	Uiso	? Au
Au8	1.0	0.334406	0.333293	0.265464	Uiso	? Au
Au9	1.0	0.335411	0.666695	0.264755	Uiso	? Au
Au10	1.0	0.166670	0.000000	0.172300	Uiso	? Au
Au11	1.0	0.166670	0.333330	0.172300	Uiso	? Au
Au12	1.0	0.166670	0.666670	0.172300	Uiso	? Au
Au13	1.0	0.083421	0.166857	0.265014	Uiso	? Au
Au14	1.0	0.085330	0.500031	0.264643	Uiso	? Au
Au15	1.0	0.083495	0.833160	0.265031	Uiso	? Au
Au16	1.0	0.416670	0.166670	0.172300	Uiso	? Au
Au17	1.0	0.416670	0.500000	0.172300	Uiso	? Au
Au18	1.0	0.416670	0.833340	0.172300	Uiso	? Au
Au19	1.0	0.250000	0.166670	0.078170	Uiso	? Au
Au20	1.0	0.250114	0.166704	0.359948	Uiso	? Au
Au21	1.0	0.250000	0.500000	0.078170	Uiso	? Au
Au22	1.0	0.250007	0.500065	0.359922	Uiso	? Au
Au23	1.0	0.250000	0.833340	0.078170	Uiso	? Au
Au24	1.0	0.250157	0.833198	0.359835	Uiso	? Au
Au25	1.0	0.500000	0.000000	0.078170	Uiso	? Au
Au26	1.0	0.499983	-0.000106	0.359946	Uiso	? Au
Au27	1.0	0.500000	0.333330	0.078170	Uiso	? Au
Au28	1.0	0.500383	0.333770	0.362455	Uiso	? Au
Au29	1.0	0.500000	0.666670	0.078170	Uiso	? Au
Au30	1.0	0.500035	0.665099	0.361571	Uiso	? Au
Au31	1.0	0.833371	0.000190	0.264995	Uiso	? Au
Au32	1.0	0.833335	0.334026	0.265257	Uiso	? Au
Au33	1.0	0.833708	0.665925	0.265370	Uiso	? Au
Au34	1.0	0.666670	0.000000	0.172300	Uiso	? Au
Au35	1.0	0.666670	0.333330	0.172300	Uiso	? Au
Au36	1.0	0.666670	0.666670	0.172300	Uiso	? Au
Au37	1.0	0.583153	0.166924	0.265431	Uiso	? Au
Au38	1.0	0.583857	0.499550	0.265463	Uiso	? Au
Au39	1.0	0.583777	0.832846	0.265426	Uiso	? Au
Au40	1.0	0.916670	0.166670	0.172300	Uiso	? Au
Au41	1.0	0.916670	0.500000	0.172300	Uiso	? Au
Au42	1.0	0.916670	0.833340	0.172300	Uiso	? Au
Au43	1.0	0.750000	0.166670	0.078170	Uiso	? Au
Au44	1.0	0.750049	0.166705	0.359945	Uiso	? Au
Au45	1.0	0.750000	0.500000	0.078170	Uiso	? Au
Au46	1.0	0.748462	0.499760	0.361834	Uiso	? Au

Au47	1.0	0.750000	0.833340	0.078170	Uiso	? Au
Au48	1.0	0.749996	0.833298	0.359923	Uiso	? Au
C1	1.0	0.005721	0.005562	0.494006	Uiso	? C
C2	1.0	0.127244	0.251571	0.493617	Uiso	? C
C3	1.0	0.000662	0.173312	0.493547	Uiso	? C
C4	1.0	0.131095	0.417650	0.493134	Uiso	? C
C5	1.0	0.005917	0.505366	0.492940	Uiso	? C
C6	1.0	0.136607	0.749907	0.493623	Uiso	? C
C7	1.0	0.009949	0.671566	0.493340	Uiso	? C
C8	1.0	0.131575	0.917513	0.494182	Uiso	? C
C9	1.0	0.255964	0.997815	0.494306	Uiso	? C
C10	1.0	0.375367	0.245977	0.492988	Uiso	? C
C11	1.0	0.252392	0.164091	0.493855	Uiso	? C
C12	1.0	0.260609	0.494290	0.492156	Uiso	? C
C13	1.0	0.390681	0.748260	0.492307	Uiso	? C
C14	1.0	0.264493	0.662630	0.492607	Uiso	? C
C15	1.0	0.382020	0.914452	0.493835	Uiso	? C
C16	1.0	0.504766	0.000264	0.493588	Uiso	? C
C17	1.0	0.502360	0.162863	0.492961	Uiso	? C
C18	1.0	0.635058	0.760219	0.492206	Uiso	? C
C19	1.0	0.632630	0.922681	0.493537	Uiso	? C
C20	1.0	0.755309	0.008504	0.493819	Uiso	? C
C21	1.0	0.872868	0.260607	0.492424	Uiso	? C
C22	1.0	0.746858	0.174828	0.492429	Uiso	? C
C23	1.0	0.876598	0.428864	0.491791	Uiso	? C
C24	1.0	0.884892	0.758953	0.493649	Uiso	? C
C25	1.0	0.762085	0.677003	0.492106	Uiso	? C
C26	1.0	0.881338	0.925176	0.494151	Uiso	? C
N1	1.0	0.379209	0.405677	0.491499	Uiso	? N
N2	1.0	0.620898	0.247297	0.491335	Uiso	? N
N3	1.0	0.516539	0.676087	0.490128	Uiso	? N
N4	1.0	0.758575	0.517599	0.489834	Uiso	? N
Fe1	1.0	0.569137	0.461594	0.486731	Uiso	? Fe
H1	1.0	0.397308	0.516618	0.581644	Uiso	? H
H2	1.0	0.593619	0.365846	0.596254	Uiso	? H
O1	1.0	0.459680	0.516567	0.611320	Uiso	? O
O2	1.0	0.583470	0.472567	0.584316	Uiso	? O

Fe1&Au NPs

#===
=

CRYSTAL DATA

#---

```
_
data_VESTA_phase_1

_chemical_name_common                  'Fe1&Au NPs '
_cell_length_a                         17.043400
_cell_length_b                         9.840000
_cell_length_c                         15.000000
_cell_angle_alpha                      90.000000
_cell_angle_beta                       90.000000
_cell_angle_gamma                      90.000000
_cell_volume                           2515.605851
_space_group_name_H-M_alt              'P 1'
_space_group_IT_number                 1

loop_
_space_group_symop_operation_xyz
   'x, y, z'

loop_
   _atom_site_label
   _atom_site_occupancy
   _atom_site_fract_x
   _atom_site_fract_y
   _atom_site_fract_z
   _atom_site_adp_type
   _atom_site_U_iso_or_equiv
   _atom_site_type_symbol
   C1         1.0     0.087511     0.181630     0.139316     Uiso  ? C
   C2         1.0     0.213774     0.055732     0.138298     Uiso  ? C
   C3         1.0     0.170546     0.180260     0.140001     Uiso  ? C
   C4         1.0     0.047491     0.056164     0.137485     Uiso  ? C
   C5         1.0     0.335229     0.193608     0.132066     Uiso  ? C
   C6         1.0     0.462254     0.072243     0.120464     Uiso  ? C
   C7         1.0     0.419275     0.199221     0.124417     Uiso  ? C
   C8         1.0     0.296866     0.062560     0.133960     Uiso  ? C
   C9         1.0     0.587926     0.193239     0.113313     Uiso  ? C
   C10        1.0     0.713897     0.065484     0.116099     Uiso  ? C
   C11        1.0     0.671775     0.190885     0.113719     Uiso  ? C
   C12        1.0     0.546072     0.069260     0.115238     Uiso  ? C
   C13        1.0     0.838937     0.186762     0.123642     Uiso  ? C
   C14        1.0     0.964148     0.058613     0.133153     Uiso  ? C
   C15        1.0     0.922040     0.184242     0.130153     Uiso  ? C
   C16        1.0     0.797279     0.062763     0.120939     Uiso  ? C
   C17        1.0     0.086820     0.431432     0.139354     Uiso  ? C
```

C18	1.0	0.210753	0.304752	0.140047	Uiso	? C
C19	1.0	0.168781	0.430533	0.141020	Uiso	? C
C20	1.0	0.045732	0.306499	0.137918	Uiso	? C
C21	1.0	0.462120	0.325580	0.119315	Uiso	? C
C22	1.0	0.590190	0.441406	0.109435	Uiso	? C
C23	1.0	0.714327	0.314442	0.112879	Uiso	? C
C24	1.0	0.673293	0.439963	0.108285	Uiso	? C
C25	1.0	0.546192	0.319294	0.112756	Uiso	? C
C26	1.0	0.839082	0.436980	0.121229	Uiso	? C
C27	1.0	0.963018	0.308983	0.132839	Uiso	? C
C28	1.0	0.921878	0.435079	0.128850	Uiso	? C
C29	1.0	0.797480	0.312378	0.118858	Uiso	? C
C30	1.0	0.090409	0.680493	0.138106	Uiso	? C
C31	1.0	0.174647	0.674475	0.139210	Uiso	? C
C32	1.0	0.046564	0.557918	0.136965	Uiso	? C
C33	1.0	0.467435	0.569448	0.115263	Uiso	? C
C34	1.0	0.425905	0.695339	0.119319	Uiso	? C
C35	1.0	0.590978	0.694238	0.109795	Uiso	? C
C36	1.0	0.715024	0.564899	0.109102	Uiso	? C
C37	1.0	0.673935	0.691233	0.108240	Uiso	? C
C38	1.0	0.549665	0.568479	0.112570	Uiso	? C
C39	1.0	0.839341	0.687626	0.121054	Uiso	? C
C40	1.0	0.963625	0.559776	0.131712	Uiso	? C
C41	1.0	0.922442	0.685455	0.128395	Uiso	? C
C42	1.0	0.797784	0.562979	0.116393	Uiso	? C
C43	1.0	0.090457	0.930816	0.138361	Uiso	? C
C44	1.0	0.217604	0.800942	0.137479	Uiso	? C
C45	1.0	0.174430	0.927882	0.138725	Uiso	? C
C46	1.0	0.048628	0.806745	0.136560	Uiso	? C
C47	1.0	0.340079	0.937444	0.129611	Uiso	? C
C48	1.0	0.466037	0.819911	0.116746	Uiso	? C
C49	1.0	0.423019	0.944270	0.122460	Uiso	? C
C50	1.0	0.301785	0.806430	0.132249	Uiso	? C
C51	1.0	0.588997	0.944016	0.112655	Uiso	? C
C52	1.0	0.714687	0.815784	0.112494	Uiso	? C
C53	1.0	0.672400	0.941325	0.113434	Uiso	? C
C54	1.0	0.548918	0.818793	0.111562	Uiso	? C
C55	1.0	0.839338	0.937218	0.123492	Uiso	? C
C56	1.0	0.964822	0.809054	0.132080	Uiso	? C
C57	1.0	0.922654	0.934446	0.129826	Uiso	? C
C58	1.0	0.797787	0.813253	0.118554	Uiso	? C
N1	1.0	0.424942	0.450920	0.120421	Uiso	? N
N2	1.0	0.291280	0.311992	0.136985	Uiso	? N
N3	1.0	0.211848	0.549196	0.140458	Uiso	? N

```
   N4          1.0     0.345834     0.688045     0.128531    Uiso   ? N
   Fe1         1.0     0.318859     0.499949     0.135302    Uiso   ? Fe
   Au1         1.0     0.579647     0.577194     0.284427    Uiso   ? Au
   Au2         1.0     0.625654     0.790429     0.389348    Uiso   ? Au
   Au3         1.0     0.725740     0.568361     0.360368    Uiso   ? Au
   Au4         1.0     0.485707     0.658193     0.426705    Uiso   ? Au
   Au5         1.0     0.617928     0.575870     0.513414    Uiso   ? Au
   Au6         1.0     0.624401     0.359861     0.400500    Uiso   ? Au
   Au7         1.0     0.471376     0.404013     0.351180    Uiso   ? Au
```

H2O2-Fe1&Au NPs

```
#======================================================================
=
# CRYSTAL DATA
#----------------------------------------------------------------------
-
data_VESTA_phase_1

_chemical_name_common                  'H2O2-Fe1&Au NPs'
_cell_length_a                         17.043400
_cell_length_b                         9.840000
_cell_length_c                         15.000000
_cell_angle_alpha                      90.000000
_cell_angle_beta                       90.000000
_cell_angle_gamma                      90.000000
_cell_volume                           2515.605851
_space_group_name_H-M_alt              'P 1'
_space_group_IT_number                 1

loop_
_space_group_symop_operation_xyz
   'x, y, z'

loop_
   _atom_site_label
   _atom_site_occupancy
   _atom_site_fract_x
   _atom_site_fract_y
   _atom_site_fract_z
   _atom_site_adp_type
   _atom_site_U_iso_or_equiv
   _atom_site_type_symbol
   C1          1.0     0.086606     0.179253     0.132757    Uiso   ? C
```

C2	1.0	0.212775	0.053385	0.131760	Uiso	? C
C3	1.0	0.169621	0.177818	0.133422	Uiso	? C
C4	1.0	0.046582	0.053809	0.130992	Uiso	? C
C5	1.0	0.334056	0.191379	0.127475	Uiso	? C
C6	1.0	0.461190	0.070320	0.116611	Uiso	? C
C7	1.0	0.418294	0.197079	0.121220	Uiso	? C
C8	1.0	0.295757	0.060460	0.127831	Uiso	? C
C9	1.0	0.586966	0.191167	0.112733	Uiso	? C
C10	1.0	0.712881	0.063382	0.114586	Uiso	? C
C11	1.0	0.670748	0.188805	0.113534	Uiso	? C
C12	1.0	0.545084	0.067296	0.112726	Uiso	? C
C13	1.0	0.837927	0.184623	0.120984	Uiso	? C
C14	1.0	0.963233	0.056500	0.127550	Uiso	? C
C15	1.0	0.921102	0.182144	0.125650	Uiso	? C
C16	1.0	0.796277	0.060620	0.118425	Uiso	? C
C17	1.0	0.085845	0.429138	0.132675	Uiso	? C
C18	1.0	0.209941	0.302392	0.134024	Uiso	? C
C19	1.0	0.167867	0.428573	0.134276	Uiso	? C
C20	1.0	0.044841	0.304137	0.131656	Uiso	? C
C21	1.0	0.461173	0.323431	0.117530	Uiso	? C
C22	1.0	0.589165	0.439346	0.109916	Uiso	? C
C23	1.0	0.713163	0.312390	0.113813	Uiso	? C
C24	1.0	0.671992	0.437941	0.110344	Uiso	? C
C25	1.0	0.545256	0.317343	0.112539	Uiso	? C
C26	1.0	0.837969	0.434868	0.119779	Uiso	? C
C27	1.0	0.962084	0.306835	0.127775	Uiso	? C
C28	1.0	0.920901	0.432991	0.124970	Uiso	? C
C29	1.0	0.796358	0.310249	0.118050	Uiso	? C
C30	1.0	0.089435	0.678142	0.131191	Uiso	? C
C31	1.0	0.173553	0.672106	0.132512	Uiso	? C
C32	1.0	0.045611	0.555571	0.130570	Uiso	? C
C33	1.0	0.466184	0.566897	0.114099	Uiso	? C
C34	1.0	0.424367	0.693655	0.114935	Uiso	? C
C35	1.0	0.589895	0.692364	0.107495	Uiso	? C
C36	1.0	0.713649	0.562912	0.111445	Uiso	? C
C37	1.0	0.672659	0.689329	0.107861	Uiso	? C
C38	1.0	0.548821	0.566443	0.113020	Uiso	? C
C39	1.0	0.838293	0.685502	0.118834	Uiso	? C
C40	1.0	0.962630	0.557670	0.126818	Uiso	? C
C41	1.0	0.921465	0.683406	0.124142	Uiso	? C
C42	1.0	0.796626	0.560857	0.116546	Uiso	? C
C43	1.0	0.089504	0.928379	0.131546	Uiso	? C
C44	1.0	0.216502	0.798662	0.131089	Uiso	? C
C45	1.0	0.173468	0.925277	0.132028	Uiso	? C

C46	1.0	0.047683	0.804450	0.129923	Uiso	? C
C47	1.0	0.338941	0.935353	0.123417	Uiso	? C
C48	1.0	0.464816	0.818073	0.111616	Uiso	? C
C49	1.0	0.421832	0.942270	0.116976	Uiso	? C
C50	1.0	0.300703	0.804548	0.126013	Uiso	? C
C51	1.0	0.587935	0.942003	0.109803	Uiso	? C
C52	1.0	0.713562	0.813813	0.111023	Uiso	? C
C53	1.0	0.671354	0.939369	0.111499	Uiso	? C
C54	1.0	0.547828	0.816877	0.107575	Uiso	? C
C55	1.0	0.838355	0.935057	0.120015	Uiso	? C
C56	1.0	0.963878	0.806961	0.126542	Uiso	? C
C57	1.0	0.921726	0.932378	0.124857	Uiso	? C
C58	1.0	0.796710	0.811143	0.116297	Uiso	? C
N1	1.0	0.423920	0.448780	0.117891	Uiso	? N
N2	1.0	0.290200	0.309604	0.132818	Uiso	? N
N3	1.0	0.210587	0.546904	0.134064	Uiso	? N
N4	1.0	0.344658	0.686471	0.122984	Uiso	? N
Fe1	1.0	0.318127	0.498504	0.138755	Uiso	? Fe
Au1	1.0	0.546920	0.600265	0.282190	Uiso	? Au
Au2	1.0	0.610470	0.789559	0.395490	Uiso	? Au
Au3	1.0	0.701110	0.577132	0.336909	Uiso	? Au
Au4	1.0	0.482892	0.630928	0.449100	Uiso	? Au
Au5	1.0	0.626430	0.567507	0.515092	Uiso	? Au
Au6	1.0	0.638701	0.348059	0.408338	Uiso	? Au
Au7	1.0	0.483224	0.382817	0.363638	Uiso	? Au
H1	1.0	0.380525	0.647604	0.338417	Uiso	? H
H2	1.0	0.371170	0.449964	0.310783	Uiso	? H
O1	1.0	0.323126	0.631245	0.328101	Uiso	? O
O2	1.0	0.322670	0.497306	0.284648	Uiso	? O

H2O-Fe1&Au NPs

#==
=

CRYSTAL DATA

#--
-

data_VESTA_phase_1

_chemical_name_common	'H2O-Fe1&Au NPs'
_cell_length_a	17.043400
_cell_length_b	9.840000
_cell_length_c	15.000000
_cell_angle_alpha	90.000000

```
_cell_angle_beta                    90.000000
_cell_angle_gamma                   90.000000
_cell_volume                        2515.605851
_space_group_name_H-M_alt           'P 1'
_space_group_IT_number              1

loop_
_space_group_symop_operation_xyz
   'x, y, z'

loop_
   _atom_site_label
   _atom_site_occupancy
   _atom_site_fract_x
   _atom_site_fract_y
   _atom_site_fract_z
   _atom_site_adp_type
   _atom_site_U_iso_or_equiv
   _atom_site_type_symbol
   C1    1.0   0.087391   0.181758   0.133765   Uiso   ? C
   C2    1.0   0.213527   0.056069   0.133664   Uiso   ? C
   C3    1.0   0.170368   0.180508   0.134863   Uiso   ? C
   C4    1.0   0.047363   0.056297   0.131900   Uiso   ? C
   C5    1.0   0.334862   0.193953   0.130118   Uiso   ? C
   C6    1.0   0.461992   0.072640   0.119388   Uiso   ? C
   C7    1.0   0.419087   0.199504   0.123666   Uiso   ? C
   C8    1.0   0.296543   0.063047   0.130347   Uiso   ? C
   C9    1.0   0.587699   0.193338   0.113903   Uiso   ? C
   C10   1.0   0.713649   0.065471   0.115228   Uiso   ? C
   C11   1.0   0.671524   0.190892   0.114103   Uiso   ? C
   C12   1.0   0.545841   0.069460   0.114853   Uiso   ? C
   C13   1.0   0.838720   0.186740   0.121315   Uiso   ? C
   C14   1.0   0.964019   0.058705   0.128045   Uiso   ? C
   C15   1.0   0.921899   0.184321   0.125976   Uiso   ? C
   C16   1.0   0.797060   0.062747   0.118818   Uiso   ? C
   C17   1.0   0.086669   0.431585   0.133883   Uiso   ? C
   C18   1.0   0.210696   0.305073   0.135862   Uiso   ? C
   C19   1.0   0.168670   0.431128   0.135933   Uiso   ? C
   C20   1.0   0.045635   0.306621   0.132575   Uiso   ? C
   C21   1.0   0.461957   0.325777   0.119296   Uiso   ? C
   C22   1.0   0.589938   0.441620   0.111363   Uiso   ? C
   C23   1.0   0.713965   0.314470   0.114271   Uiso   ? C
   C24   1.0   0.672777   0.440050   0.111433   Uiso   ? C
   C25   1.0   0.545995   0.319530   0.113851   Uiso   ? C
```

C26	1.0	0.838753	0.437011	0.120460	Uiso	? C
C27	1.0	0.962899	0.309039	0.128337	Uiso	? C
C28	1.0	0.921674	0.435098	0.125613	Uiso	? C
C29	1.0	0.797159	0.312382	0.118546	Uiso	? C
C30	1.0	0.090161	0.680615	0.132499	Uiso	? C
C31	1.0	0.174281	0.674625	0.134268	Uiso	? C
C32	1.0	0.046388	0.557938	0.131691	Uiso	? C
C33	1.0	0.467124	0.569184	0.117460	Uiso	? C
C34	1.0	0.425390	0.695938	0.118967	Uiso	? C
C35	1.0	0.590645	0.694343	0.110001	Uiso	? C
C36	1.0	0.714416	0.564960	0.112893	Uiso	? C
C37	1.0	0.673333	0.691315	0.110170	Uiso	? C
C38	1.0	0.549688	0.568659	0.115031	Uiso	? C
C39	1.0	0.839072	0.687635	0.119552	Uiso	? C
C40	1.0	0.963419	0.559801	0.127564	Uiso	? C
C41	1.0	0.922258	0.685487	0.124746	Uiso	? C
C42	1.0	0.797406	0.563000	0.117492	Uiso	? C
C43	1.0	0.090237	0.930922	0.132730	Uiso	? C
C44	1.0	0.217239	0.801233	0.133175	Uiso	? C
C45	1.0	0.174189	0.928002	0.133706	Uiso	? C
C46	1.0	0.048430	0.806884	0.130952	Uiso	? C
C47	1.0	0.339763	0.937924	0.126324	Uiso	? C
C48	1.0	0.465732	0.820279	0.115514	Uiso	? C
C49	1.0	0.422702	0.944651	0.120421	Uiso	? C
C50	1.0	0.301489	0.807118	0.128956	Uiso	? C
C51	1.0	0.588704	0.944108	0.112181	Uiso	? C
C52	1.0	0.714298	0.815814	0.112625	Uiso	? C
C53	1.0	0.672104	0.941383	0.112907	Uiso	? C
C54	1.0	0.548664	0.818847	0.110884	Uiso	? C
C55	1.0	0.839134	0.937217	0.120478	Uiso	? C
C56	1.0	0.964651	0.809154	0.127176	Uiso	? C
C57	1.0	0.922511	0.934533	0.125280	Uiso	? C
C58	1.0	0.797483	0.813285	0.117151	Uiso	? C
N1	1.0	0.424738	0.451008	0.120189	Uiso	? N
N2	1.0	0.290991	0.312244	0.135400	Uiso	? N
N3	1.0	0.211392	0.549478	0.136129	Uiso	? N
N4	1.0	0.345539	0.688888	0.126901	Uiso	? N
Fe1	1.0	0.318919	0.500782	0.141508	Uiso	? Fe
Au1	1.0	0.544156	0.581884	0.290276	Uiso	? Au
Au2	1.0	0.593295	0.789414	0.397160	Uiso	? Au
Au3	1.0	0.698567	0.591230	0.343787	Uiso	? Au
Au4	1.0	0.476251	0.613991	0.457391	Uiso	? Au
Au5	1.0	0.624380	0.573837	0.519116	Uiso	? Au
Au6	1.0	0.638546	0.356834	0.408910	Uiso	? Au

Au7	1.0	0.481693	0.364542	0.374033	Uiso	? Au
H1	1.0	0.355982	0.596900	0.306261	Uiso	? H
H2	1.0	0.364646	0.442977	0.312112	Uiso	? H
O1	1.0	0.328630	0.513732	0.287870	Uiso	? O
